# *In silico* analysis of class I adenylate-forming enzymes reveals family and group-specific conservations

**DOI:** 10.1371/journal.pone.0203218

**Published:** 2018-09-04

**Authors:** Louis Clark, Danielle Leatherby, Elizabeth Krilich, Alexander J. Ropelewski, John Perozich

**Affiliations:** 1 Department of Biology, Franciscan University of Steubenville, Steubenville, OH, United States of America; 2 Pittsburgh Supercomputing Center, Carnegie Mellon University, Pittsburgh, PA, United States of America; Wake Forest University, UNITED STATES

## Abstract

Luciferases, aryl- and fatty-acyl CoA synthetases, and non-ribosomal peptide synthetase proteins belong to the class I adenylate-forming enzyme superfamily. The reaction catalyzed by the adenylate-forming enzymes is categorized by a two-step process of adenylation and thioesterification. Although all of these proteins perform a similar two-step process, each family may perform the process to yield completely different results. For example, luciferase proteins perform adenylation and oxidation to produce the green fluorescent light found in fireflies, while fatty-acyl CoA synthetases perform adenylation and thioesterification with coenzyme A to assist in metabolic processes involving fatty acids. This study aligned a total of 374 sequences belonging to the adenylate-forming superfamily. Analysis of the sequences revealed five fully conserved residues throughout all sequences, as well as 78 more residues conserved in at least 60% of sequences aligned. Conserved positions are involved in magnesium and AMP binding and maintaining enzyme structure. Also, ten conserved sequence motifs that included most of the conserved residues were identified. A phylogenetic tree was used to assign sequences into nine different groups. Finally, group entropy analysis identified novel conservations unique to each enzyme group. Common group-specific positions identified in multiple groups include positions critical to coordinating AMP and the CoA-bound product, a position that governs active site shape, and positions that help to maintain enzyme structure through hydrogen bonds and hydrophobic interactions. These positions could serve as excellent targets for future research.

## Introduction

Class I adenylate-forming enzymes (also termed the ANL superfamily [1]) include aryl- and acyl-CoA synthetases, fatty acid-AMP ligases, methylmalonyl-CoA synthetases, the adenylation domain of non-ribosomal peptide synthetases, and luciferases. They represent one class in a superfamily of enzymes that carry out adenylation, the activation of a carboxylate substrate through the formation of an AMP-intermediate. A nucleophile then attacks the intermediate, releasing the AMP [2]. These enzymes perform a wide variety of functions such as fatty acid metabolism, detoxification of halogenated aromatic compounds, antibiotic synthesis, and bioluminescence [3–7]. Two other classes of adenylate-forming enzymes exist: class II includes aminoacyl-tRNA synthetases and class III includes NRPS-independent siderophore synthesis enzymes. Neither class II or III enzymes have homologous structures to class I enzymes [2]. All three classes are dependent on Mg^2+^ [8], although the number of these ions used varies among the enzymes [9].

Previous sequence analysis of these enzymes revealed several highly conserved areas including the P-loop, the linker (L) motif, adenine (A) motif, gate (G) motif and the magnesium-binding site. The P-loop coordinates the phosphate-binding site allowing for cleavage of ATP by the substrate, forming an AMP-intermediate and a pyrophosphate leaving group. The L motif joins the larger amino-terminal and smaller carboxy-terminal domains, allowing for movement of these domains depending on the bound substrate. The A motif contains critical residues for binding the adenine moiety in ATP/AMP. The G motif includes the gate residue that controls substrate access to the fatty acid binding site in long chain fatty acyl-CoA synthetases (LACSs) [3]. The magnesium-binding site coordinates the magnesium ion that neutralizes the charge of ATP as well as the pyrophosphate leaving group, stabilizing each [2].

**Fig 1:**
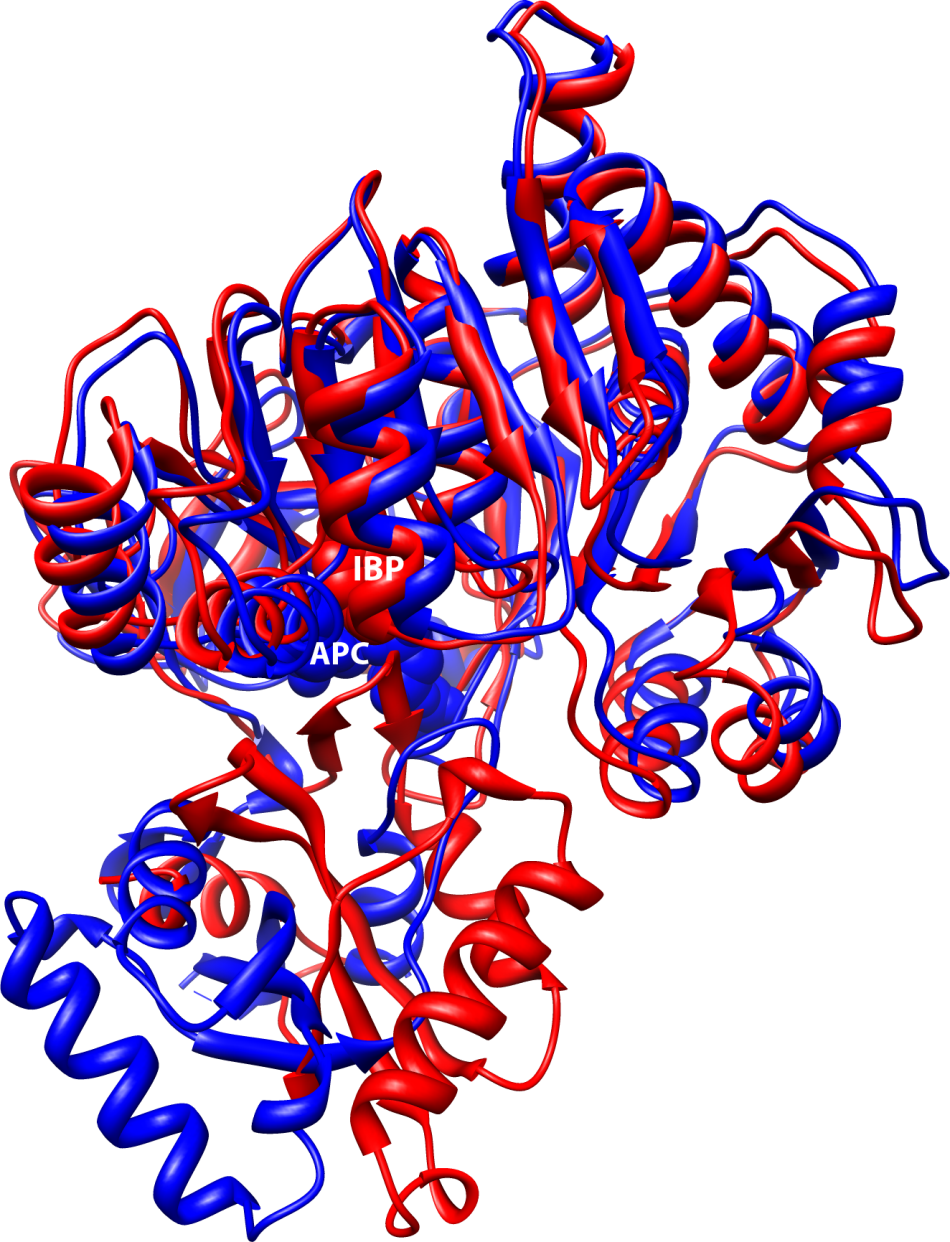
Carboxy-terminal domain rotation in human MACS, aligned using the j-FATCAT rigid algorithm. The adenylation conformation is shown in blue (PDB ID: 3DAY) with APC, an ATP analog, bound. The thioesterification conformation is shown in red (PDB ID: 2WD9) with ibuprofen (IBP) bound. The amino-terminal domain is well aligned in both conformations (top), but it is the carboxy-terminal domain (bottom) that moves via the flexible linker motif.

These enzymes have a conserved structure of two domains that undergo changes in orientation depending on the molecule bound in the active site (termed domain alternation), resulting in one large functional domain that can selectively catalyze adenylation or thioesterification reactions [1] (Fig 1). In human medium chain fatty acyl-CoA synthetase (MACS) the enzyme begins the reaction in the adenylate conformation. Using a bi-uni-uni-bi ping-pong mechanism [1] the fatty acid substrate and ATP bind to this conformation. The pyrophosphate of ATP prevents conformational change through interactions with the P-loop and a conserved lysine (Lys557 in human MACS). Formation of the fatty acyl-AMP intermediate and release of the pyrophosphate allows a 140° rotation of the flexible linker and a repositioning of the carboxy-terminal domain to form the thioesterification conformation. The acid anhydride bond between the acyl group and AMP provides the energy for the thioesterification reaction. In this thioesterification conformation CoA can bind to react to form fatty acyl-CoA and release the AMP [5]. This domain alternation appears unique to these adenylate-forming enzymes [1].

One type of class I adenylate-forming enzyme is fatty-acyl CoA synthetase (ACS). There are several subtypes based upon preferred fatty acid substrate length. These include short-chain ACS (SACS, EC 6.2.1.1) which prefer substrates with 2–4 carbons, medium-chain ACS (MACS, EC 6.2.1.2) which prefer substrates with 4–12 carbons and long-chain ACS (LACS, EC 6.2.1.3) which prefer substrates with 12–22 carbons. These enzymes are critical to fatty acid metabolism by activating fatty acids through esterifying CoA to the carboxyl group to form fatty acyl-CoAs, via the adenylate intermediate [3,5]. Acetyl-CoA synthetase is a SACS present in all organisms that converts acetate to acetyl-CoA to help ensure sufficient levels of this critical metabolite [10]. Mammalian LACSs also influence various cellular activities including protein transport and acylation [11,12] and cell signaling [13], among others. In *Candida albicans* LACSs are necessary for cellular metabolism during the formation of biofilms [14]. A study [15] has also shown that expression of a LACS in *Streptomyces coelicolor* is required for antibiotic production. Conversely, disruption of LACS function has decreased the virulence of several bacterial species, including *Vibrio cholerae* [16], *Salmonella enterica* serovar Typhi [17] and *Mycobacterium tuberculosis* [18]. Mutations in LACSs in *Haemophilus parasuis* also decreased survival and increased antibiotic sensitivity [19].

Several other class I enzymes also act through adenylate adducts to generate a thioester CoA product. Methylmalonyl-CoA synthetase (MMCS) converts malonate to malonyl-CoA, likely during malonate conversion to acetyl-CoA. Malonate appears to be an important growth substrate in nitrogen-fixing nodules associated with plant roots [20]. Aryl-CoA ligases (ACLs) catalyze the joining of aromatic compounds to CoA. For example, the well-studied 4-chlorobenzoate:CoA ligase (CBL) assists in aromatic degradation by converting 4-chlorobenzoate and ATP to 4-chlorobenzoyl-CoA and AMP through an adenylated intermediate [4,21,22]. In plants aryl-CoA ligases are involved in the synthesis of flavonoids, anthocyanins and lignin [23].

Luciferases (EC 1.13.12.7) in fireflies and luminous beetles also share a common structure with these other adenylate-forming enzymes. In the phenomenon of bioluminescence luciferases react luciferin with ATP to form an adenylated intermediate. Unlike most of the superfamily that would then proceed to a thioesterification reaction, the luciferyl-AMP reacts with O_2_ in an oxidative decarboxylation to form AMP, CO_2_ and emit a photon of light, typically in the yellow-green wavelength [24]. A S286N mutation in *Luciola cruciata* luciferase shifts the emission wavelength to red [25]. However, under anaerobic conditions the luciferyl-AMP intermediate can react with CoA to form luciferyl-CoA [26]. In fact, luciferases appear to also act as LACSs, preferring substrates such as linolenic and arachidonic acids [27]. In addition, a single mutation of Ser345 in *Agrypnus binodulus* ACS allowed for luminescent activity [28]. Bioluminescence occurs in several organisms including bacteria, dinoflagellates, jellyfish, crustaceans, insects and fish. It is believed that bioluminescence may have convergently evolved up to thirty times [29,30].

Another family member is the adenylate-forming domain of non-ribosomal peptide synthetases (NRPSs). Bacteria and fungi possess NRPSs to synthesize antibiotic peptides such as cyclosporin A, gramicidin S [7], enterobactin [31], tyrocidine [32] and acinetobactin [33]. NRPSs have multiple components which each add a single amino acid to the antibiotic peptide. Each module has an adenylation domain that shares homology to class I adenylate-forming enzymes. This domain takes the amino acid and ATP and forms an amino acyl-AMP intermediate. For the thioesterification step, a peptidyl carrier protein (PCP) domain, instead of free CoA, is used to form a thioester to the amino acid and release AMP. This amino acyl moiety is finally added to the peptide using an unrelated condensation domain, without the involvement of ribosomes [1,34]. A study of NRPS mutants in *Pseudomonas aeruginosa* suggests the NRPS product cyclodipeptides affect bacterial quorum sensing and root development in plants [35]. Fatty acid-AMP ligases (FAALs) form a fatty acyl-AMP intermediate from a fatty acid and ATP, similar for ACSs. However, in a process analogous to NRPSs the fatty acyl group is transferred to an acyl carrier protein component of the enzyme polyketide synthase. This pathway helps to generate lipids associated with virulence in organisms like *Mycobacterium tuberculosis* [36,37].

A large number of sequences and representative tertiary structures are available for each type of class I adenylate-forming enzyme. There has not been an extensive study that has compared these enzymes. The goal of this research was to align a large number of protein sequences for each homologue. We then attempted to identify and confirm the conserved structural and functional roles of residues and sequence motifs in all of these enzymes. Phylogenetic analysis was used to examine family relationships and identify enzyme groups for further analysis. Group entropy analysis and other methods indicated group-specific conservations for each enzyme homologue, identifying key residue positions that may help to determine the unique function of each enzyme.

## Materials and methods

The procedure used here was analogous to the procedure we previously published [38,39]. The project initially began by obtaining the amino acid sequences and tertiary structures of *Luciola cruciata* luciferase (PDB ID: 2D1R), *Brevibacillus brevis* gramicidin synthase phenylalanine-activating domain (PDB ID: 1AMU), *Thermus thermophilus* long chain fatty acyl-CoA synthetase (PDB ID: 1V25), human medium chain fatty acyl-CoA synthetase (PDB ID: 2WD9 & 3DAY), *Alcaligenes* 4-chlorobenzoyl:CoA ligase (CBL, PDB ID: 3CW9), *Salmonella enterica* acetyl-CoA synthetase (PDB ID: 1PG3), *Rhodopseudomonas palustris* methylmalonyl-CoA synthetase (PDB ID: 4FUQ), *Methanosarcina acetivorans* acyl-adenylate synthetase (PDB ID: 3ETC), *Legionella pneumophila* fatty acid-AMP ligase (PDB ID: 3KXW), *E*. *coli* fatty acid-AMP ligase (PDB ID: 3PBK), *Acinetobacter baumannii* BasE (PDB ID: 3O82) and *Mycobacterium tuberculosis* FadD10 long chain fatty acyl-CoA ligase (PDB ID: 4IR7) from the RCSB Protein Data Bank. Each sequence was then used to perform a PSI-Blast [40] search of the non-redundant protein database at the National Center for Biotechnology Information (NCBI). A total of 374 amino acid sequences of class I adenylate-forming enzymes were collected with percent identities ranging from 99% to 12%. These sequences were initially aligned using T-Coffee [41]. To improve alignment quality, the alignment was manually adjusted using tertiary structure comparison of all structures using MAPSCI (http://www.geom-comp.umn.edu/mapsci/) [42] and through the RCSB PDB Protein Comparison Tool-jFATCAT method [43,44] of pairs of structures as a guide. The alignment editor used was GENEDOC [45]. Conservations within the alignment were analyzed for structural or functional significance. Molecular visualization and distance calculations were performed using RASMOL [46]. Salt bridges were identified as amino and carboxylate groups that were less than 3.0Å in distance apart. Hydrogen bonds were identified as hydrophilic groups that were less than or equal to 3.3Å in distance apart. Hydrophobic interactions were identified as nonpolar atoms less than or equal to 4.5Å in distance apart. Molecular graphics were generated using Chimera [47]. Torsional angles were determined using MolProbity [48]. Analysis of conserved sequence motifs was facilitated by MEME program [49], and these motifs were searched against a protein database using MAST [50]. Group entropy analysis (GEnt) [51] was performed to compare SACS, ACL, FAAL, FadD10, LACS, MACS, Luciferase, MMCS and NRPS groups to each other. Evolutionary trace (http://mordred.bioc.cam.ac.uk/~jiye/evoltrace/evoltrace.html) [52,53] was also performed on the entire alignment. Protein residue conservation prediction (http://compbio.cs.princeton.edu/conservation/score.html) [54] was performed on subalignments of each of the nine groups identified. Each algorithm was used using combinations of both possible backgrounds (BLOSUM62 and SwissProt) and seven possible matrices (BLOSUM62, BLOSUM35, BLOSUM40, BLOSUM45, BLOSUM50, BLOSUM80 and BLOSUM100) distributed with the program. Scores presented for Shannon Entropy and Property Entropy represent the top 25 scoring residues. For Relative Entropy and JS Divergence, residue positions reported were predicted by all distributions used. For VN Entropy and Sum of Pairs analyses, residues reported were predicted using all seven scoring matrices (BLOSUM62, BLOSUM35, BLOSUM40, BLOSUM45, BLOSUM50, BLOSUM80 and BLOSUM100) distributed with the program.

The PHYLIP suite of programs was used to generate the phylogenetic tree [55]. First, the alignment was trimmed using TrimAl [56]. 400 Bootstrapped data sets of the trimmed alignment were then generated using the SEQBOOT program. Next, distances for the data sets were determined by the PROTDIST program using the Jones-Taylor-Thornton matrix. Phylogenetic trees for each data set were generated using the NEIGHBOR program. Lastly, the unrooted consensus tree was generated using the CONSENSE program. The tree graphic was generated using FigTree (available at http://tree.bio.ed.ac.uk/software/figtree). A parsimony tree was generated using 75 bootstrapped datasets using the PROTPARS program, followed by CONSENSE [55].

## Results

### Structure and residue conservations

**Fig 2:**
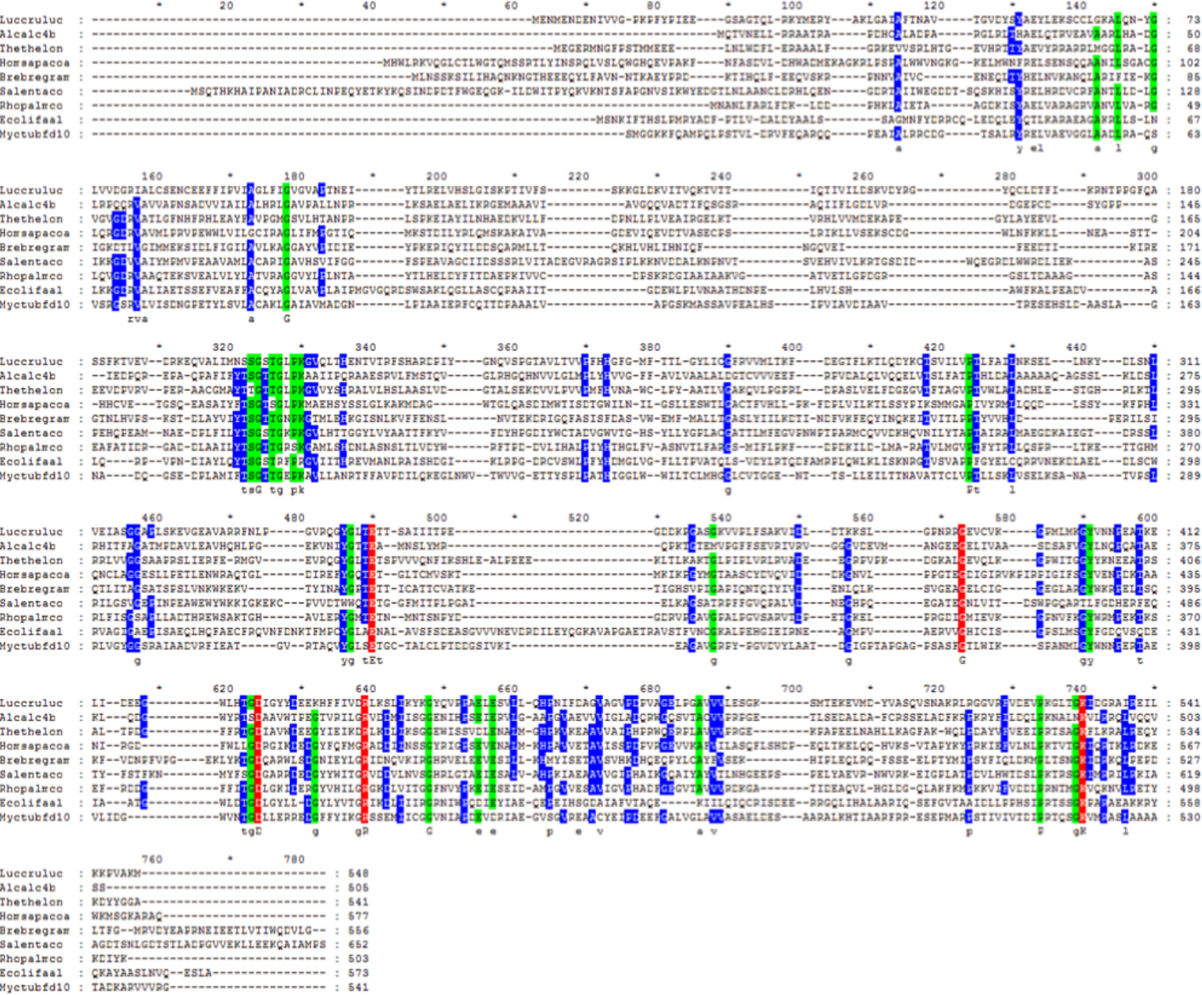
Summary alignment showing a representative sequence for each group of class I adenylate-forming enzymes. Sequences include *Luciola cruciata* luciferase (Luccruluc), *Alcaligenes* 4-chlorobenzoyl-CoA ligase (Alcalc4b) as an ACL, *Thermus thermophilus* LACS (Thethelon), human MACS (Homsapacoa), *Brevibacillus brevis* gramicidin synthase phenylalanine-activating domain (Brebregram) as an NRPS, *Salmonella enterica* acetyl-CoA synthetase (Salentaco) as a SACS, *Rhodopseudomonas palustris* MMCS (Rhopalmco), *E*. *coli* FAAL (Ecolifaal) and *Mycobacterium tuberculosis* FadD10 long chain fatty acyl-CoA ligase (Myctubfd10). The entire alignment, which contains 374 protein sequences, is found in S1 File. Residue positions are colored based upon their conservation in the entire alignment as follows: red = 100% conserved, green = 80–99% conserved, and blue = 60–79% conserved. Indel (gap) positions from the entire alignment (S1 File) are retained to allow correlation with index position numbers (numbers shown above the alignment columns) that are noted within the text.

**Fig 3:**
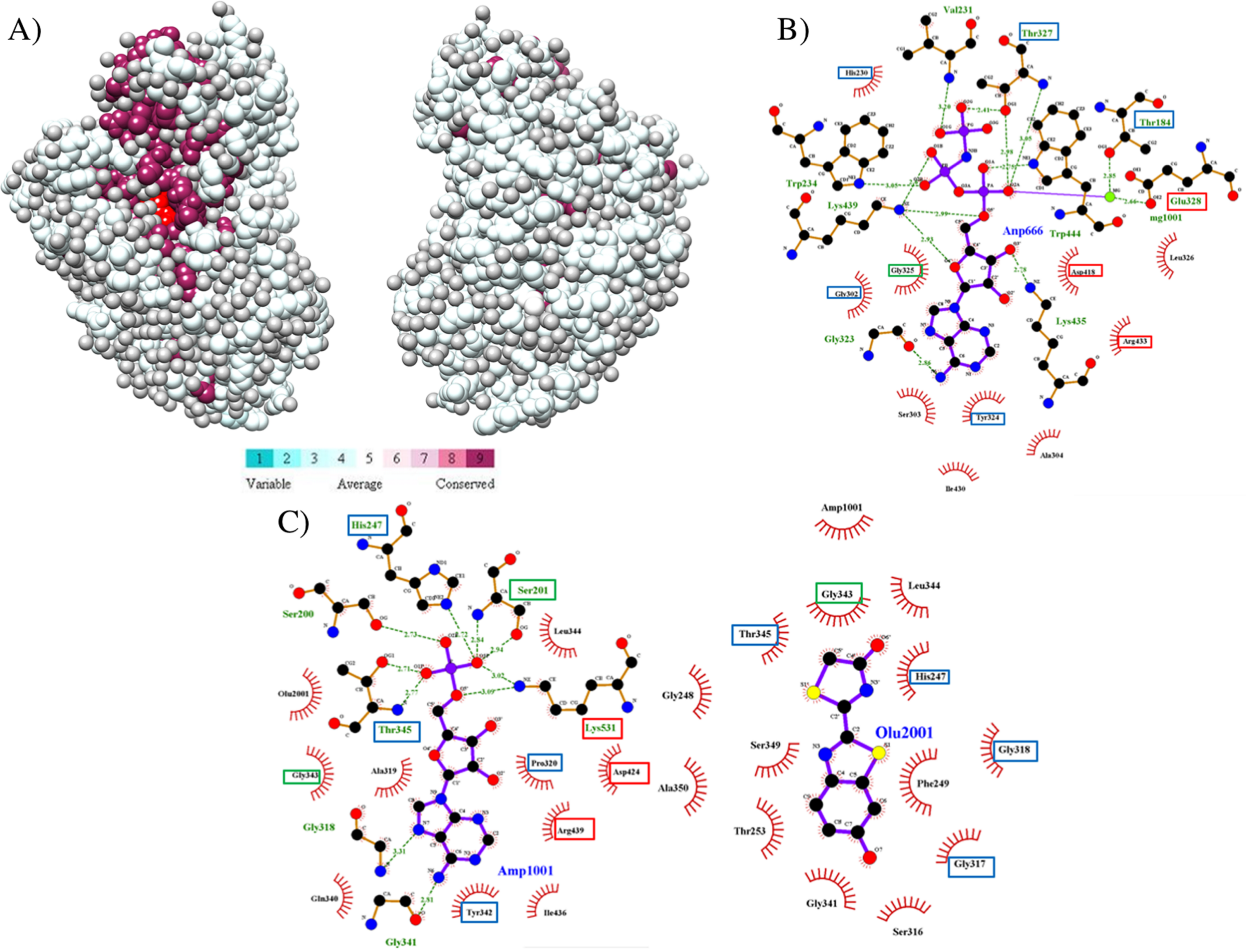
Conserved residues in class I adenylate-forming enzymes. (A) Evolutionarily conserved residue positions as determined by the CONSURF program [57]. Shown are front and back views (180° rotation) of *Luciola cruciata* luciferase (PDB ID: 2D1R). The bound AMP molecule (red) is shown. Residue conservation scale is from the CONSURF website. Note how most conserved positions surround the AMP in the active site. (B) Ligplot [58] diagram highlighting residues in the active site that contact the bound ANP (ANP666) in *T*. *thermophilus* LACS (PDB ID: 1V25). Boxes surrounding the residue names indicate conservation from the alignment: red = 100% conserved, green = 80–99% and blue = 60–79%. (C) Ligplot diagram highlighting residues in the active site that contact the bound AMP (AMP1001) and oxyluciferin (Olu2001) ligands in *L*. *cruciata* luciferase (PDB ID: 2D1R), also using color coding to highlight residue conservation.

A total of 374 amino acid sequences from the class I adenylate-forming superfamily were aligned (Fig 2), guided by tertiary structural alignment. The entire alignment can be found in S1 File. Above each amino acid position column is an index number, which is numbered concurrently from the beginning of the alignment; these index numbers will be used to reference each position throughout this manuscript. The sequences used included 49 aryl-CoA ligases (ACLs), 84 luciferase sequences, 42 LACSs, 66 MACSs, 53 NRPSs, 25 acetyl-CoA synthetases (SACSs), 31 MMCSs, 17 FAALs, and 7 mycobacterial FadD10 fatty-acyl CoA ligase sequences. Five residue positions were invariant among all 374 sequences: Glu328{490}, Gly384{573}, Asp418{624}, Arg433{639} and Lys524{740} (residue positions are in *Thermus thermophilus* LACS (sequence Thethelon), unless otherwise noted, with alignment index positions in curly brackets). A total of 22 additional residues were conserved in at least 80% of the sequences aligned and 56 more residues conserved in at least 60%. A summary of the conserved residue interactions is found in Table 1. The locations of these evolutionary conservations were also visualized using the CONSURF program [57] (Fig 3A). Highly conserved residues in the family are clustered around the active site, which is the pocket in the enzyme where the substrates are bound, while the least conserved residues are located on the enzyme surface. Residue functions were analyzed using the *Thermus thermophilus* LACS (sequence Thethelon, PDB ID: 1V25) structure, with exceptions using the *Luciola cruciata* firefly luciferase (sequence Luccruluc, PDB ID: 2D1R) structure. Residues within the active site in both *T*. *thermophilus* LACS and *L*. *cruciata* luciferase are shown in Fig 3B and 3C. *T*. *thermophilus* LACS structure was chosen for analysis as it had ligands, ANP and Mg^2+^, bound in its active site and also had a substrate modeled to allow for atomic distances to be measured and functions to be interpreted. In addition, the function of several conserved residues had already been proposed [3]. *L*. *cruciata* luciferase structure was also chosen as it also had ligands bound in its active site to assist analysis and as it was the initial structure used in beginning the project.

**Table 1:** Interactions of selected conserved residues in adenylate-forming enzymes.

Index	Residue Identity^a^	Conservation	Residue Interactions^b^
142	Met61	87%	CE is 3.8Å from Trp21{93}.CZ2; CE is 4.2Å from Pro170{305}.CD
145	Leu64	81%	CD2 is 4.5Å from Val75{157}.CG1
157	Val75	68%	CG1 is 4.5Å from Leu64{145}.CD2
321	Tyr183	61%	OH is 2.7Å from His117{206}.ND1; OH is 3.3Å from Ala118{207}.N
322	Thr184	70%	OG1 is 2.9Å from Mg^2+^
323	Thr185	94% Ser	CG2 is 3.8Å from Leu106{195}.CD2; OG1 is 3.3Å from a water molecule, which is in turn 2.4Å from ANP.O3G
324	Gly186	97%	CA is 4.8Å from Mg^2+^
325	Thr187	72%	CB is 6.4Å from Mg^2+^; CA is 4.4Å from Leu437{643}.CD2
326	Thr188	93%	OG1 is 4.3Å from Leu437{643}.CD1; CG2 is 4.9Å from Ser446{652}.OG
327	Gly189	93%	Turn in P-Loop
329	Pro191	88%	CG is 4.3Å from Tyr113{202}.CE1; CG is 4.8Å from Glu110{199}.CG
330	Lys192	97%	NZ is 3.3Å from Thr187{325}.OG; NZ is 3.1Å from Thr188{326}.O
424	Pro275	95%	CG is 3.6Å from Val300{455}.CG2; CG is 4.0Å from Ser303{458}.CB; CB is 4.4Å from Pro306{461}.CD
456	Gly301	67%	O is 3.4Å from Pro275{424}.CD; CA is 4.9Å from Gly323{485}.CA; within 4.5Å of myristoyl group of substrate [3]
457	Gly302	67%	N is 3.4Å from ANP.N7; O is 3.3Å from AMP.O4’
486	Tyr324	73%	CB is 4.1Å from ANP.C6; within 4.5Å of myristoyl group of substrate [3]
487	Gly325	93%	CA is 3.0Å from Pro331{493}.O; CA is 3.7Å from Thr329{491}.OG1; within 4.5Å of myristoyl group of substrate [3]
489	Thr327	78%	N is 2.9Å from ANP.O2A; OG1 are 2.9Å from AMP.O2A; within 4.5Å of myristoyl group of substrate [3]
490	Glu328	100%	OE2 is 2.7Å from Mg^2+^
573	Gly384	100%	CA is 3.7Å from Val368{548}.CG1
591	Tyr397	93%	OH is 2.6Å from Glu328{490}.OE1
624	Asp418	100%	OD1 is 2.6Å from ANP.O2’; OD2 is 2.7Å from ANP.O3’
632	Gly426	85%	CA is 3.8Å Leu30{103}.CD2 (Intersubunit)
639	Arg433	100%	NH2 is 2.7Å from Leu437{643}.O; NH2 is 2.7Å from Glu475{682}.OE1; NH1 is 3.0Å from a water molecule, which is in turn 2.9Å from ANP.O2’
655	Asp449	85% Glu	OD1 is 3.5Å from Ser446{652}.OG
657	Glu451	97%	OE2 is 2.7Å from Lys527{743}.NZ; OE2 is 2.8Å from Val465{672}.N
686	Ala479	85%	CB is 3.9Å from Val465{672}.CG2
734	Pro518	99%	Pro525{734}.CG is 4.1Å from Ile537{746}.CG1 (2D1R); Pro525{734}.CD is 4.2Å from Ile540{749}.CD1 (2D1R)
740	Lys524	100%	Lys531{740}.NZ IS 3.0Å from AMP.O3P (2D1R)

^a^ Residue identity in Thethelon.

^b^ Distances measured in PDB structure 1V25, unless otherwise noted.

Among the conserved residues (Table 1), Thr184{322} and Glu328{490} coordinate the bound magnesium cofactor [3]. In CBL the hydroxyl of Thr161{322} (sequence Alcalc4b) also hydrogen bonds to the α-phosphate on AMP [21]. Site-directed mutagenesis of both residues severely impacted enzymatic activity (Table 2).

**Table 2:** Site-directed mutagenesis studies of conserved and group-specific residues in adenylate-forming enzymes.

Index	Residue Identity	Mutation	Molecule	Reference	Result of Mutation
69	Val4	Phe	*E*.*coli* FadD LACS	76	Increased growth rates with hexanoate and octanoate, but not oleate; 4-fold decrease in K_cat_/K_m_ for oleate
70	Trp5	Leu	*E*.*coli* FadD LACS	76	Increased growth rates with hexanoate and octanoate, but not oleate; 4-fold decrease in K_cat_/K_m_ for oleate
74	Tyr9	His	*E*.*coli* FadD LACS	76	Increased growth rates with hexanoate and octanoate, but not oleate; 2-fold increase in K_cat_/K_m_ for octanoate; 20% decrease in K_cat_/K_m_ for oleate
321	Tyr213	Ala	*E*. *coli* FadD ACS	62	No detectable activity
322	Thr161	Ala	CBL	22	2,000-fold decrease in K_cat_/K_m_ for both 4-chlorobenzoate and ATP; 4,000-fold decrease in k_obs_
322	Thr214	Ala	*E*. *coli* FadD ACS	62	90% decrease in V_max_ and K_cat_/K_m_; No change in K_m_ for ATP
324	Gly163	Ile	CBL	4	1,000-fold decrease in overall rate; 4,000-fold decrease in CBA-AMP formation
324	Gly216	Ala	*E*. *coli* ACS	62	75% decrease in V_max_; 70% decrease in K_cat_/K_m_; No change in K_m_ for ATP
325	Thr217	Ala	*E*. *coli* ACS	62	73% decrease in V_max_; 93% decrease in K_cat_/K_m_; 4-fold increase in K_m_ for ATP
327	Gly166	Ile	CBL	4	14-fold decrease in overall rate; 60-fold decrease in CBA-AMP formation
327	Gly219	Ala	*E*. *coli* FadD ACS	62	42% decrease in V_max_; 27% increase in K_cat_/K_m_; 46% decrease in K_m_ for ATP
329	Pro168	Ala	CBL	4	No detectable activity
330	Lys169	Met	CBL	4	4-fold decrease in overall rate; 5-fold decrease in CBA-AMP formation
330	Lys186	Arg	TycA NRPS	59	Less than 1% activity
330	Lys222	Ala	*E*. *coli* ACS	62	65% decrease in V_max_; 92% decrease in K_cat_/K_m_; 3.4-fold increase in K_m_ for ATP
330	Lys172	Ala	FadD13 ACS	61	63.5% decrease in activity; 10- fold increase in the K_m_ for ATP
373	His207	Ala	CBL	22	500-fold decrease in K_cat_/K_m_ with 4-chlorobenzoate; 90-fold decrease in k_obs_
375	Phe247	Ser	*P*. *pyralis* Luciferase	82	Increased light production with aminoluciferin
381	Thr251	Ser	*P*. *pyralis* Luciferase	82	Increased light production with aminoluciferin
461	Gln338	Arg	*E*.*coli* FadD LACS	76	Increased growth rates with hexanoate and octanoate, but not oleate; 2.5-fold increase in K_cat_/K_m_ for octanoate
486	Tyr304	Phe	CBL	22	100% of wild type activity; No change in K_cat_/K_m_ with 4-chlorobenzoate, CoA and ATP
489	Thr307	Ala	CBL	22	100-fold decrease in K_cat_/K_m_ with 4-chlorobenzoate; 60-fold decrease in k_obs_
490	Glu306	Gln	CBL	4	50-fold decrease in overall rate; 50-fold decrease in CBA-AMP formation; 18-fold increase in 4-CBA K_m_
490	Glu361	Ala	*E*. *coli* FadD ACS	62	No detectable activity
501	Asp372	Gly	*E*.*coli* FadD LACS	76	Increased growth rates with hexanoate and octanoate, but not oleate; 35% decrease in K_cat_/K_m_ for oleate
533	His376	Arg	*E*.*coli* FadD LACS	76	Increased growth rates with hexanoate and octanoate, but not oleate; 35% decrease in K_cat_/K_m_ for oleate
623	Gly437	Ala	*E*. coli ACS	60	50–70% decreased activity; No change in substrate preference
624	Asp385	Ala	CBL	22	500-fold decrease in K_cat_/K_m_ for both 4-chlorobenzoate and ATP; 300-fold decrease in k_obs_
624	Asp438	Ala	*E*. *coli* ACS	60	No detectable activity
624	Asp401	Ser	TycA NRPS	59	90% decrease in activity
632	Gly446	Ala	*E*. *coli* ACS	60	Nearly stopped activity with decanoate and oleate, but not myristoate
633	Phe447	Ser	*E*.*coli* FadD LACS	76	Increased growth rates with hexanoate and octanoate, but not oleate; 50% decrease in K_cat_/K_m_ for octanoate; 55% decrease in K_cat_/K_m_ for oleate
637	Val451	Ala	*E*.*coli* FadD LACS	76	Increased growth rates with hexanoate and octanoate, but not oleate; 3.5-fold increase in K_cat_/K_m_ for octanoate; 15% decrease in K_cat_/K_m_ for oleate
639	Arg453	Ala	*E*. *coli* ACS	60	Essentially abolished enzymatic activity
639	Arg400	Ala	CBL	22	100-fold decrease in K_cat_/K_m_ with 4-chlorobenzoate and ATP; 600-fold decrease in K_cat_/K_m_ with CoA; 160-fold decrease in k_obs_
646	Ser404	Ala	FadD13 ACS	61	39% decrease in activity; 6- fold increase in the K_m_ for CoA
648	Gly409	Leu	CBL	21	Loss of activity only during the thioesterification step
657	Glu457	Lys	*L*. *mingrelica* luciferase	63,64	Shift to red emission
740	Lys492	Ala	CBL	22	500-fold decrease in K_cat_/K_m_ with 4-chlorobenzoate; 600-fold decrease in k_obs_
740	Lys487	Ala	FadD13 ACS	61	96.3% decrease in activity

Several conserved residues interact with the ATP/AMP coenzyme (Table 1). Gly302{457} and Tyr324{486} interact with the adenine moiety [3]. A mutation of Tyr304{486} in CBL to phenylalanine did not alter enzyme function, as phenylalanine could still ring stack with the adenine ring [22] (Table 2). Asp418{624} coordinates both the 2’ and 3’ ribose hydroxyls, while Arg433{639}, which is found in the linker motif, also interacts with the 2’ hydroxyl through a water molecule [3]. Mutations of both residues severely hindered enzymatic activity [22,59,60] (Table 2). In addition, Gly302{457} interacts with the 4’ hydroxyl involved in the hemiacetal bond [3]. In CBL the adenine ring of the substrate-AMP adduct is located between the equivalent glycine (Gly281{457}) and Thr283{459}. It has also been suggested that a glycine at index 457 in CBL probably keeps the phosphopantetheine tunnel open [21]. Thr327{489} forms two hydrogen bonds of the α-phosphate on AMP [3]. Mutagenesis of the equivalent threonine (Thr307{489}) in CBL caused a significant reduction in catalytic efficiency with the 4-chlorobenzoate substrate [22] (Table 2). Thr185{323} interacts through a water molecule with the γ–phosphate of ANP. In CBLs the main chain nitrogen and side chain hydroxyl of Thr165{323} also interact with the γ–phosphate of ATP [22]. Lastly, while Lys524{740} lacked structural coordinates in the *T*. *thermophilus* LACS structure, Lys531{740} in the *L*. *cruciata* luciferase structure coordinates the α-phosphate of AMP [25]. The equivalent residue in CBL (Lys492{740}) lies close to and may react with the carboxylate group of the substrate in the adenylation conformation, with a significant decrease in rate for this part of the reaction seen in a K492A mutant (Table 2). This lysine rotates into the solvent in the thioesterification conformation [22]. The binding of the lysine at index 740 to ATP was also supported by mutagenesis in *Mycobacterium tuberculosis* FadD13 ACS [61] (Table 2). Thus, the majority of the invariantly conserved residues coordinate the AMP moiety and the critical Mg^2+^ ion, functions shared by all family members.

Four conserved residues (Table 1) line the myristoyl substrate pocket of the *T*. *thermophilus* LACS structure: Gly301{456}, Tyr324{486}, Gly325{487} and Thr327{489} [3]. The conserved glycine at index 487 lies at a location that is a tryptophan in SACS (Trp414{487} in *S*. *enterica* SACS, sequence Salentaco). This bulkier residue likely results in a shorter fatty acid substrate preference in SACS, while a glycine would allow for longer fatty acids to bind to MACSs and LACSs [5]. The carbonyl oxygen of the equivalent glycine in gramicidin synthase (Gly324{487}, sequence Brebregram) hydrogen bonds to the amino group of the phenylalanine substrate [7].

Other conserved residues act to maintain enzyme folding through hydrophobic interactions, identified as less than or equal to 4.5Å in distance (Table 1). These include Met61{142}, Leu64{145}, Val75{157}, Thr187{325}, Thr188{326}, Pro191{329}, Pro275{424}, Gly301{456}, Gly325{487}, Gly384{573}, Gly426{632}, Ala479{686} and Pro518{734}. Leu64{145} and Val75{157} interact with each other. The three conserved prolines, Pro191{329}, Pro275{424} and Pro518{734}, are found in turns in the *T*. *thermophilus* LACS structure.

Several other conserved residues may also help to maintain enzyme structure through hydrogen bond or salt bridge formation (Table 1). The hydroxyl of Tyr183{321} forms a hydrogen bond to His117{206}. A Y213A mutant at index 321 in *E*. *coli* ACS resulted in no detectable activity [62] (Table 2). Lys192{330} lies at the end of the P loop and its side chain amine interacts with the carbonyl oxygen of another conserved residue, Thr188{326}, and also lies close to the hydroxyl of Thr187{325}. Mutagenesis of the lysine at index 330 and the threonine at index 325 both significantly hindered activity (Table 2). The hydroxyl of Tyr397{591} forms a hydrogen bond with the side chain carboxylate of the invariant Glu328{490}. The side chain guanidinium of Arg433{639} forms a hydrogen bond to the carbonyl oxygen of Leu437{643} and a salt bridge to the side chain carboxylate of Glu475{682}. Mutation of the arginine at index 639 (Arg400) in CBL indicates the importance of a salt bridge with Asp402{641} to stabilize the thioesterification conformation [22] (Table 2). Asp449{655} lies at a position that is always an acidic residue, with glutamate being is 85% conserved in the entire alignment. The side chain carboxylate of Asp449{655} lies close to the hydroxyl of Ser446{652} in *T*. *thermophilus* LACS. Glu416{655} in CBL forms a salt bridge to Lys474{722} and a hydrogen bond to the main chain nitrogen of His413{652}. Lastly, the side chain carboxylate of Glu451{657} forms a hydrogen bond to the main chain nitrogen of Val465{672} and a salt bridge to the side chain amine of Lys527{743}. An E457K mutation in *Luciola mingrelica* luciferase at index 657 (Table 2) caused a strong red shift in emission color, and suggested that rigidity in the carboxy-terminal domain is important for green emission in luciferases [63,64].

Eleven of the 27 residues conserved in at least 80% of sequences in the entire alignment were glycine residues: Gly68{150}, Gly96{178}, Gly186{324}, Gly189{327}, Gly325{487}, Gly358{538}, Gly384{573}, Gly417{623}, Gly426{632}, Gly442{648}, and Gly523{739}. The overrepresentation of glycines among the highly conserved residues is due to their critical role in protein structure in turns or where the lack of a side chain is necessary. This phenomenon occurs in other enzyme families, such as aldehyde dehydrogenases [65], alcohol dehydrogenases [66,67], arginases [68] and NDP-sugar dehydrogenases [38]. Seven conserved glycines (Gly68{150}, Gly96{178}, Gly186{324}, Gly189{327}, Gly358{538}, Gly426{632}, and Gly442{648}) lie at turns in the enzyme structure, as seen within the 1V25 *T*. *thermophilus* LACS structure. Of those seven conserved glycines found in turns, all but Gly186{324} had positive phi angles, which is common in glycines found in turns [69]. In CBL Gly409{648}, which is part of the previously identified motif A8 [70], lines the tunnel for binding the phosphopantetheine portion of CoA. Mutation of this residue to leucine resulted in activity loss only during the thioesterification step [21] (Table 2). Three other glycines (Gly325{487}, Gly384{573}, and Gly417{623}) are found in beta strands. Mutation of the glycine at index 623 in *E*. coli ACS (Gly437) significantly reduced activity, but did not change substrate preference [60] (Table 2). Next, Gly426{632} is found at the dimer interface of the *T*. *thermophilus* LACS structure, making hydrophobic contact with Leu30{103} from the neighboring subunit. Mutation of the glycine at index 632 in *E*. coli ACS (Gly446) significantly reduced activity for two of the three fatty acid substrates tested [60] (Table 2). Three highly conserved residues, Gly202{324}, Gly205{327}, and Pro207{329}, are found in the P-loop of *L*. *cruciata* luciferase which suggests that these residues may play critical structural roles for the P-loop. In human MACS Gly223{324} lines the pyrophosphate-binding pocket [5]. Mutations in all three of these residues in the P-loop severely inhibited enzymatic activity (Table 2).

### Conserved motifs

**Fig 4:**
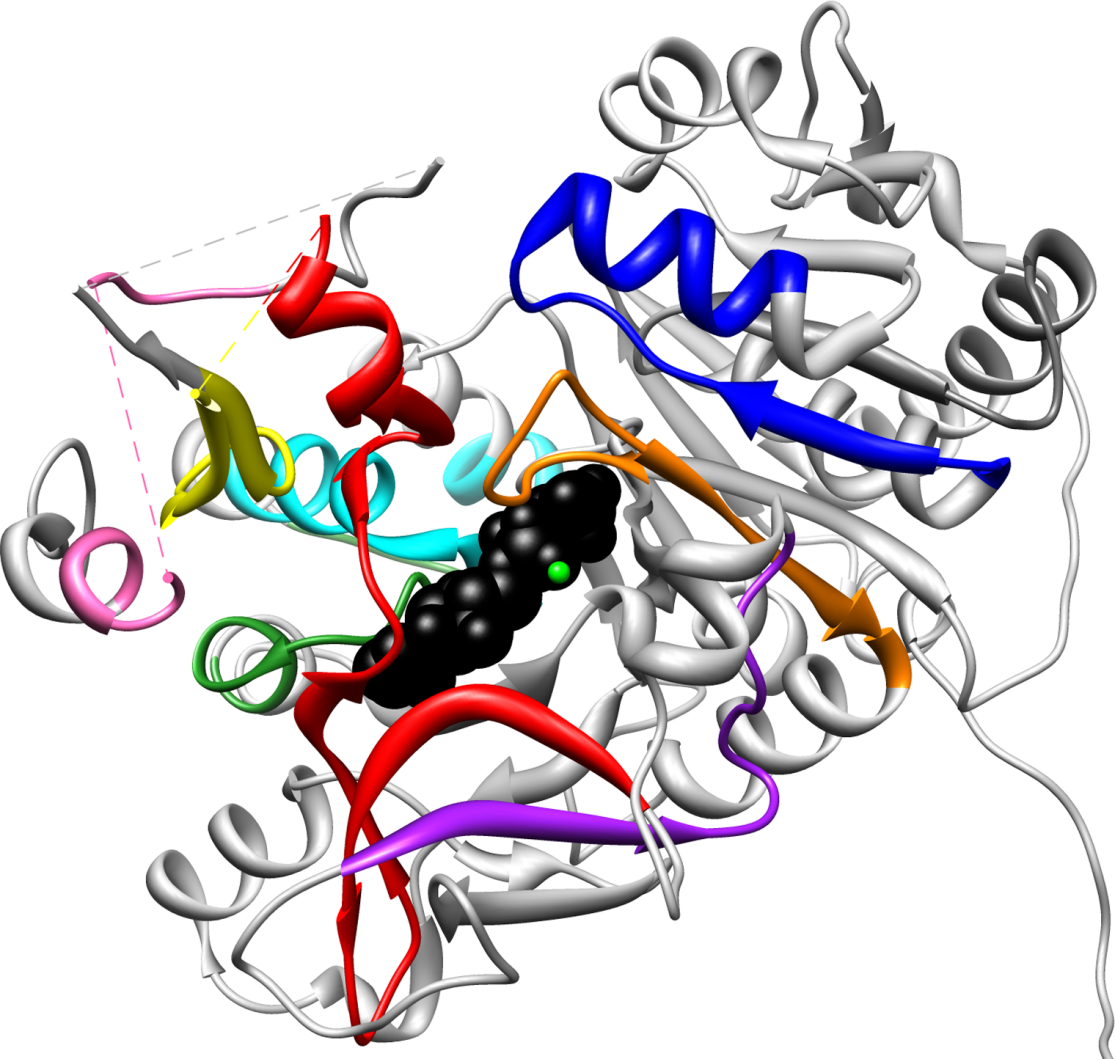
Conserved motifs found in the monomer of *Thermus themophilus* LACS (PDB ID: 1V25). The bound ANP molecule (black) and magnesium ion (green) are shown in the active site. Motifs 1 (red), 2 (pink), 3 (orange), 4 (yellow), 7 (dark green), 9 (cyan) and 10 (blue) line the active site.

The ten most conserved sequence motifs were statistically identified using the MEME program [49] (Table 3). Four of the five fully conserved residues cluster into three of the conserved motifs. Several of these motifs correlate to motifs previously identified specifically in the adenylation domain of NRPSs [70] (Table 3). Motif 1, which covers previous NRPS motifs A7 & A8, contains two invariant residues, Asp418{624} and Arg433{639}. Residues in motif 1 line the active site (Fig 4) and include the linker motif. Beta strands 19–22 and helix α-N comprise motif 1 (structural terminology from [3]). Motif 2 contains the fully conserved Lys524{740} and covers previous NRPS motif A10. It contains β-25 and α-P. Two highly conserved residues, Thr188{326} and Lys192{330}, are found in motif 3, which correlates to NRPS motif A3. Motif 3 lines the active site and includes the P-loop. Motif 3 has been well studied through site-directed mutagenesis (summarized in [70]), which suggest that it is critical in the adenylation step [4]. Motif 4 also lines the active site but is not present in NRPSs and FAALs, which both join the substrate to a carrier protein instead of CoA. Motif 5, which covers the previous NRPS motif A6, contains Gly384{573} which is found in β-18. This motif did not appear in the LACS, SACS, or MMCS groups. Motif 7 lines the active site but is not present in mycobacterial FadD10s.

**Table 3:** Ten most conserved sequence motifs in class I adenylate-forming enzymes.

Motif Number	Length	Motif Regular Expression	Indices	NRPS Motif ^a^
1	41	[YLF]H[TS]GD[LI][GA][YR]xDEDGY[FL][WF][IF][VT][GD]Rx[KD]D[LV]I[KI]S[GKS]G[YEF][RNQ][IV]GPAE[IVL]ESAL	620–660	A7, A8
2	24	P[RD]x[VI][EV]FVDE[LI]PK[TN][PA][ST]GKI[LD][RK][RK]ELR	724–747	A10
3	15	[TS]SG[TS]TGLPKGV[ML][LH][TS]H	322–336	A3
4	21	HPA[VI]A[ED]AAV[VI]G[VI]P[DH][PE]x[WAR]G[EQ]V[PV]	664–684	
5	21	GE[IL]C[VI][RK]xxxxxGPG[VIL][MFA]KGY[WYL]N	568–593	A6
6	21	[RK]LANALxxxLG[VIL]K[KP]GD[RV]V[AG][LVI]L	139–160	
7	21	DLSSL[RK]xLVS[GA][GA][AE][PA]LN[PK]E[VL]xE	446–466	
8	21	ExKPGSVG[KR][PV]VP[GN]V[ED]V[KR][IVL][VI]DP	531–551	
9	21	[IL][EQ]K[YE][KR][VI]Tx[LF]xG[VA]PTIYR[FA]L[LA][KQ]	412–432	
10	29	[AI]GA[VI]VVP[LI]NPRxxxxxxx[YL]TPK[ED][IL]xYR[LI]N	177–212	A2

^a^ = Domains previously identified in the adenylation domain of NRPSs [70]

One of the few motifs identified previously in NRPSs [70] that was not identified in the top ten motifs in this study was motif A5, which has a NxYGPTE sequence, covers the adenine (A) motif [3], and would be found at indices 484–490 in our alignment. Despite the fact that it is well conserved in our alignment, including the invariant Glu328{490}, it is not surrounded by additional conservations, which might have led to it not being identified here. This stretch of residues has also been suggested to be critical in the adenylation reaction [4].

The motifs identified by MEME were used to search the Uniprot database for other proteins with potential homology to class I adenylate-forming enzymes using MAST [50]. Most proteins identified by the MAST search, which returned more than 290,000 sequence hits ranging from the strongest hit with an e-score of 4.6e-114 to the weakest hit with an e-score of 10, were class I adenylate-forming enzymes. The MAST search also discovered a class I adenylate-forming enzyme that had not been included in this project, D-alanine—poly(phosphoribitol) ligase, which is also called D-alanine-D-alanyl carrier protein ligase (ACPL). An example of an ACPL is DltA D-alanine-D-alanyl carrier protein ligase from *Streptococcus pyogenes* (sp|P0DA64|DLTA_STRP3, PDB ID: 3LGX) [9], which had an e-score in the MAST search of 1.3e-24. DltA is involved in the process of adding D-alanine to lipoteichoic acids during cell wall formation in Gram-positive bacteria [9]. DltA possesses motifs 3, 7, 8, 5, 1 and 2 (in that order). In addition, structural alignment (not shown) with *T*. *themophilus* LACS (PDB ID: 1V25) showed a close match with a RMSD value of 2.79Å and a percent identity of 14.3%.

Two other proteins that came up multiple times in the MAST search results were cinnamyl alcohol dehydrogenase and phenylalanine racemase. An example of a cinnamyl alcohol dehydrogenase is from *Arabidopsis thaliana* (tr|B1GV07|B1GV07_ARATH), which had a search e-value of 2.1e-79. It possesses motifs 6, 3, 9, 7, 8, 5, 1, 4 and 2, in that order. Structural alignment of the AtCAD5 cinnamyl alcohol dehydrogenase from *Arabidopsis* (PDB ID: 2CF5) [71] with *T*. *themophilus* LACS (PDB ID: 1V25) showed some homology with a RMSD value of 3.60Å and a percent identity of 8.6%. However, cinnamyl alcohol dehydrogenases are in a different class of enzymes, oxidoreductases, and convert an alcohol to aldehyde using NADP^+^, not ATP [71]. An example of phenylalanine racemase is an ATP-hydrolyzing phenylalanine racemase from *Serratia* (tr|V3TT50|V3TT50_SERS3), which had a search e-value of 1.5e-51. It possesses motifs 10, 3, 9, 7, 8, 5, 1, 4 and 2 in that order. It is interesting to note that this is a similar pattern of motifs as found in cinnamyl alcohol dehydrogenase. There are no protein structures for phenylalanine racemases in the PDB database, but there is a N-amino acid racemase crystallized with N-acetyl-phenylalanine from *Amycolatopsis* (PDB ID: 5FJT) (to be published). Structural alignment of N-acetyl-phenylalanine from *Amycolatopsis* with *T*. *themophilus* LACS (PDB ID: 1V25) showed some structural homology with a RMSD value of 3.65Å and 6.1% percent identity. However, phenylalanine racemase is another enzyme from a different enzyme class, isomerases.

### Phylogenetic analysis

**Fig 5:**
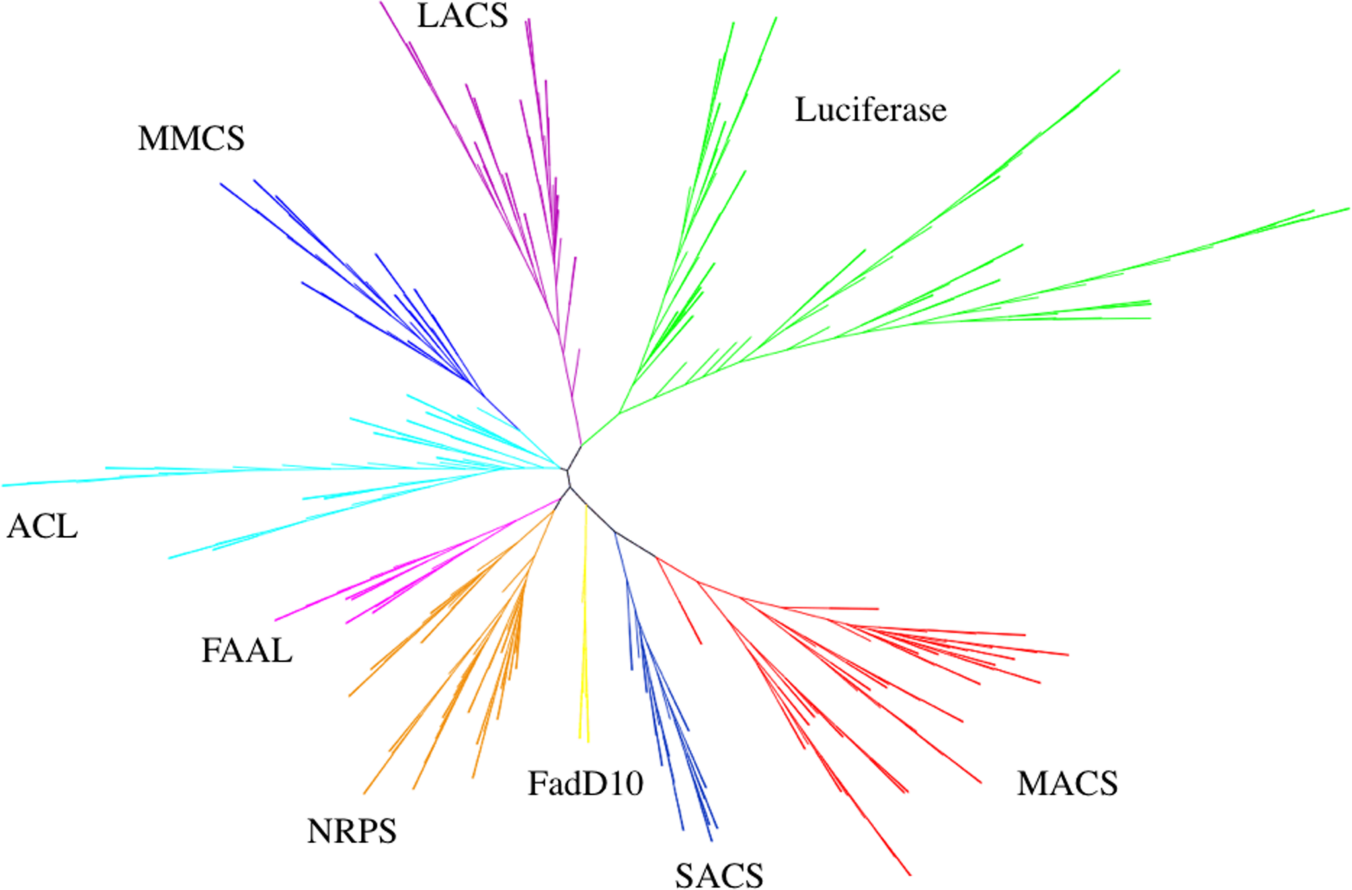
Unrooted bootstrapped neighbor-joining phylogenetic tree of class 1 adenylate-forming enzymes. Branches are color-coded based on enzyme type: green = luciferases, purple = LACS, cyan = ACL, blue = MMCS, pink = FAAL, orange = NRPS, yellow = FadD10, navy = SACS and red = MACS.

An unrooted bootstrapped phylogenetic tree of the class I adenylate-forming enzyme superfamily was generated using the neighbor-joining method (Fig 5). This method was chosen as maximum likelihood and parsimony methods are computationally prohibitive for larger datasets, and as other studies have indicated that the neighbor-joining method has yielded quality evolutionary relationships in some families [72]. In fact, a bootstrapped parsimony tree (S1 Fig) using only 75 datasets had similar group arrangements and sequence groupings to the neighbor-joining tree using 400 replicates. The neighbor-joining tree was used to assign each sequence into an appropriate group for group entropy analysis. Nine distinct groups were identified in the phylogenetic tree: Luciferases, NRPS, LACS, MACS, ACL, SACS, MMCS, FAAL and FadD10. Groups were named based upon the representative tertiary structure present in each clade, although some ACS sequence names within the group did not necessarily correlate to the group name. For example, some sequences named medium chain ACSs, when part of this larger dataset, were more homologous to the long chain ACS structure, falling within the LACS clade of the tree. It is possible some of these sequences may have been misidentified due to homology searches at the time of submission. Luciferases were most similar to LACSs. This is not unexpected as luciferases can act as long chain fatty acyl-CoA synthetases [27], and vice versa [28]. It was surprising that long-chain ACSs (LACS) were quite removed in the tree from short-chain (SACS) and medium-chain ACSs (MACS), as these fatty acyl-CoA synthetases differ solely in the length of their fatty acyl substrate. MMCSs were closely related to ACLs, but due to their substrate difference were categorized as different groups. Both groups attach substrates to CoA. Two other closely related groups were FAALs and NRPSs. Both groups attach the reaction intermediate (amino acyl-AMP in NRPSs and fatty acyl-AMP in FAALs) to a carrier protein, rather than CoA. The NRPS group contained a subgroup of fourteen 2,3-dihydroxybenzoate AMP ligase (DHB) sequences.

### Determining group-specific residues

The GEnt program [51] detects amino acid residues characteristic of an individual protein family from an alignment with other related proteins. The GEnt program utilizes the Kullback-Leibler method to calculate a divergence measure to identify covariance in protein families. GEnt calculates two entropy values, “Group Entropy” and “Family Entropy.” Group Entropy represents the degree of residue conservation at a specific position within the designated group and Family Entropy represents the degree of residue conservation at that same position within the entire alignment. This study was concerned with residues with the highest Group Entropy scores, which indicates the residues are well conserved in its group, and low Family Entropy scores, which indicates the residues are not well conserved throughout the entire alignment. These residues would indicate novel positions that contribute to the unique function and structure of each adenylate-forming homologue. The GEnt program has been used to identify critical, group-specific conservations in class 3 ALDHs [51], NDP-sugar dehydrogenases [38] and heme oxygenase homologues [39].

The Evolutionary Trace program was developed to identify critical residues in active sites and clusters of residues at functional interfaces in proteins which are unique to each group in a protein family [52,53]. In addition, six other algorithms were used to identify functional residues in each group of class I adenylate-forming enzymes: Jensen-Shannon Divergence, Property Entropy, VN Entropy, Relative Entropy, Shannon Entropy and Sum of Pairs Analysis [54]. Only residues that were identified for all combinations of backgrounds and matrices used for each algorithm were reported as results.

The GEnt results will be focused on in this manuscript for several reasons. First, GEnt has been used previously to identify group-specific residues in several families, noted above. Secondly, GEnt allows the user to define their own groups and place specific sequences in each group while analyzing the entire alignment. However, six methods used (Shannon Entropy, Property Entropy, Relative Entropy, Jansen-Shannon Divergence, VN Entropy and Sum of Pairs analyses) could not identify groups within the entire alignment, so each method had to be provided subalignments for each individual group. Thus, they tended to identify residues already conserved in the entire alignment. The Evolutionary Trace program in our analysis also tended to identify residues conserved in the entire superfamily. For example, in the Luciferase group nearly half (15 of 32) of the positions identified by Evolutionary Trace were conserved positions in at least 80% of sequences in the entire alignment. Thus, only a fraction of the residues identified by these other methods may actually be group specific. Third, there was a degree of redundancy in the positions identified by these other methods. For example, Evolutionary Trace identified 16 index positions in LACSs, all of which were also identified in Luciferases. Also, Evolutionary Trace identified the eight index positions in NRPSs, which were all also identified in LACSs and Luciferases, several of which are highly conserved in our alignment. In addition, several of the positions identified by the majority of these other methods were also identified by GEnt. Lastly, GEnt does not analyze positions in the alignment that contain predominantly gaps. For these reasons, the results for all the methods used to identify group-specific residues are summarized in S1 Dataset.

### Group-specific residues in luciferases

**Fig 6:**
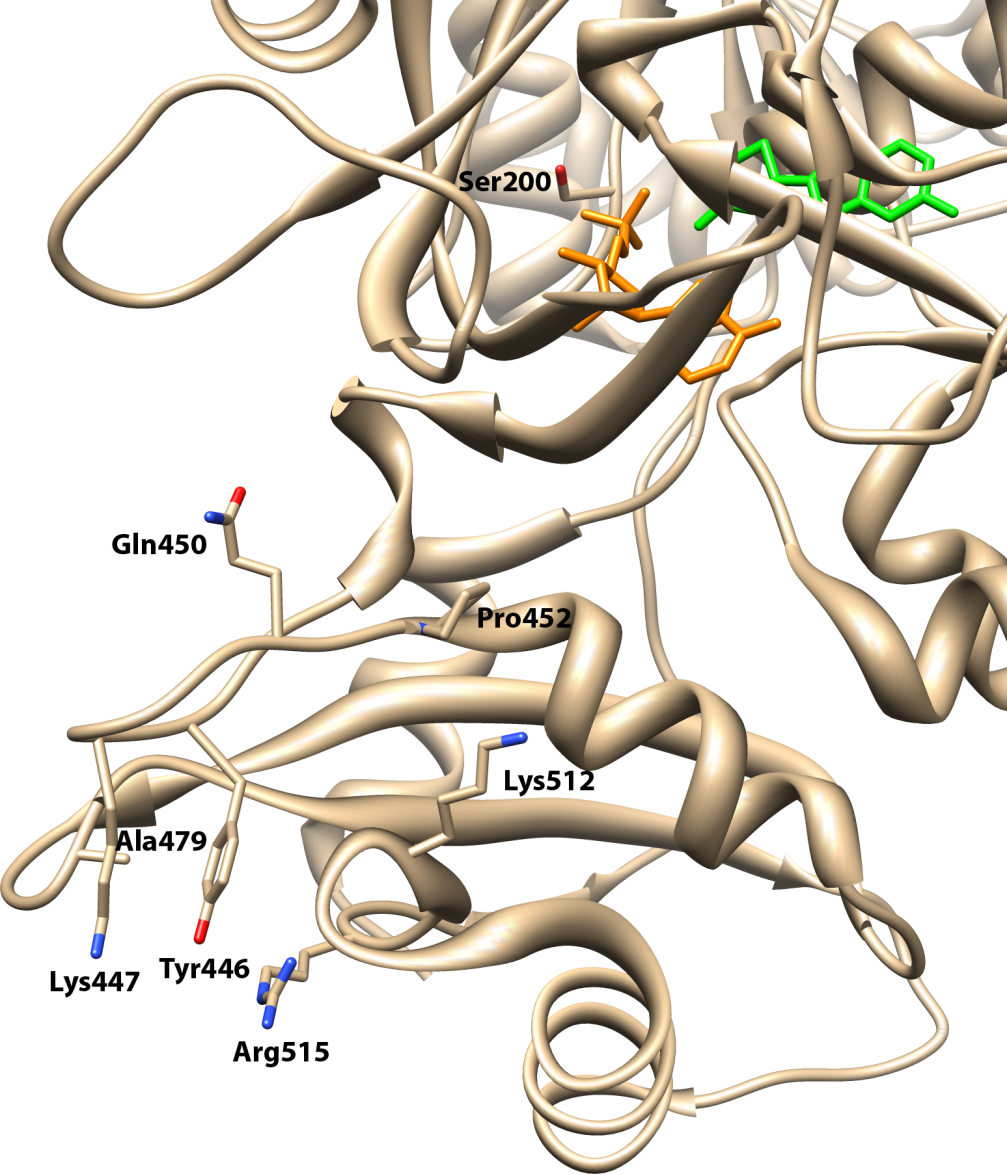
Residues with the highest Group entropy scores in luciferases. Oxyluciferin is shown in green and AMP in orange. Note how these residues cluster together in the carboxy-terminal domain (bottom).

Eight residues had the highest Group Entropy scores in the Luciferase group (Table 4). Complete GEnt results for Luciferases can be found in S2 Dataset. The combined results for all methods used to identify group-specific functional residues in Luciferases are summarized in S1 Dataset. Examination of residues was done with *L*. *cruciata* luciferase (sequence Luccruluc, PDB ID: 2D1R). One residue, Ser200{322}, hydrogen bonds to the α-phosphate of AMP. Nakatsu and colleagues [25] also showed Ser200{322} also binds to the sulfate group of the bound DLSA, which represents a substitute for AMP in the binding pocket. Pro452{652} lies at the beginning of α-18 and may be important for the structure of the loop containing Gln450{650}, also identified by GEnt in Luciferases. Two residues, Lys512{721} and Arg515{724}, form salt bridges in luciferases. The side chain amine of Lys447{647} is near the side chain hydroxyl of Tyr446{646}, but is too far for hydrogen bond formation. The remaining residues identified by GEnt (Gln450{650}, Tyr446{646} and Ala479{680}) are involved in hydrophobic interactions. Two of these residues, Tyr446{646} and Ala479{680}, contact each other. All of the highest scoring GEnt residues in Luciferases, except Ser200{322}, cluster on the surface in the carboxy-terminal domain (Fig 6). This clustering raised the question that perhaps these residues might be involved in intersubunit contact, as the *L*. *cruciata* luciferase structure is a monomer. However, analysis of the *Photinus pyralis* luciferase dimer (PDB ID: 5KYT) demonstrated that this region is not involved in dimeric contacts in that molecule [73].

**Table 4:** Group entropy analysis of luciferases.

Index	Residue Identity^a^	Group Entropy	Family Entropy	Highest Group Residue	Highest Family Residue	Conservation in Group	Residue Interactions^b^
650	Gln450	15.202	2.197	Gln	Trp	100%	OE1 is 3.9Å from Leu378{551}.CD1
721	Lys512	12.679	1.937	Lys	Tyr	99%	NZ is 2.7Å from Glu455{655}.OE1
646	Tyr446	11.885	1.246	Tyr	Tyr	87%	OH is 3.9Å from Lys447{647}.NZ; CE2 is 4.1Å from Ala511{720}.CA; CE1 is 3.5Å from Ala479{680}.CB
724	Arg515	10.160	2.391	Arg	Pro	75%	NH2 is 3.9Å from Tyr446{646}.OH; NH2 is 2.8Å from Asn510{719}.OD1
647	Lys447	9.600	1.423	Lys	Gly	98%	NZ is 3.9Å from Tyr446{646}.OH
322	Ser200	9.311	2.991	Ser	Thr	100%	OG is 2.7Åfrom AMP.O2P
652	Pro452	8.716	1.249	Pro	Tyr	53%	Turn preceding α-18
680	Ala479	8.439	1.133	Ala	Trp	93%	CB is 3.5Å from Tyr446{646}.CE1

^a^ Residue identity in Luccruluc.

^b^ Structure PDB ID: 2D1R.

Three positions, Arg218{343}, Leu286{421} and Ser347{494}, identified as lining the substrate binding site and affecting substrate specificity in *Photinus pyralis* luciferase (PDB ID: 4G36) [74], were not identified as group specific locations in luciferases in this study. In addition, none of the mutations, R214K{343}, H241K{373}, S246H{379} and H347A{488}, that caused a shift in emission wavelength of *Pyrearinus termitilluminans* luciferase [75] were identified as group specific positions in luciferases in this study. However, indices 373 and 488 were identified as group-specific positions in other groups.

### Group-specific residues in LACSs

Eight residues had the highest Group Entropy values in the long-chain fatty-acyl CoA synthetase (LACS) group (Table 5). Complete GEnt results for LACSs can be found in S3 Dataset. Group-specific residues identified in LACSs by all methods used are summarized in S1 Dataset. Examination of residues was done with *T*. *themophilus* LACS (sequence Thethelon, PDB ID: 1V25). One residue, Trp444{650}, hydrogen bonds to the α-phosphate of the AMP moiety [3]. Trp234{378} lies within 4.5Å from the myristoyl moiety of the substrate. Hisanaga and colleagues [3] refer to Trp234{378} as the “gate residue” because once ATP binds, *T*. *thermophilus* LACS transitions to a closed conformation which leads to the opening of the tryptophan gate to the fatty acid-binding tunnel. His85{167}, His100{182} and Tyr196{334} form hydrogen bonds in LACSs. His85{167} hydrogen bonds to the carbonyl oxygen of Phe80{162}, also identified by GEnt, acting to maintain enzyme folding. The remainder of the residues identified by GEnt (Phe80{162}, Trp505{721} and Ala182{320}) form hydrophobic contacts in the enzyme. Ala182{320} hydrophobically contacts Tyr196{334}, noted above.

**Table 5:** Group entropy analysis of LACSs.

Index	Residue Identity^a^	Group Entropy	Family Entropy	Highest Group Residue	Highest Family Residue	Conservation in Group	Residue Interactions^b^
650	Trp444	17.977	2.197	Trp	Trp	100%	NE1 is 2.8Å from ANP.O1A
162	Phe80	17.422	1.278	Trp	Trp	95% Trp	CD2 is 3.9Å from Pro252{398}.CB
334	Tyr196	14.276	1.513	Tyr	His	88%	OH is 2.8Å from Thr327{489}.O
721	Trp505	11.957	1.937	Trp	Tyr	95%	CZ3 is 3.6Å from Ile445{651}.CG1
182	His100	11.730	0.921	His	His	95%	ND1 is 2.8Å from Asp120{209}.OD1
320	Ala182	11.123	1.129	Cys	Cys	91% Cys	CB is 5.7Å from ANP.O1G; CB is 3.7Å from Tyr196{334}.OH
167	His85	9.463	1.658	His	Trp	95%	ND1 is 2.9Å from Phe80{162}.O
378	Trp234	9.108	1.154	Trp	Trp	100%	Within 4.5Å from myristoyl moiety of substrate [3]

^a^ Residue identity in Thethelon.

^b^ Structure PDB ID: 1V25.

A previous study [60] identified a signature sequence for ACSs, which in our alignment (Fig 2) would cover indices 607–641 and would comprise part of motif 1 identified here. This stretch contains several highly conserved residues, including Gly417{623}, Asp418{624}, Gly426{632} and Arg433{639}. However, none of the residues identified here as group-specific for LACSs are found in this region.

An additional note is that a mutagenesis study [76] was performed on *E*.*coli* FadD LACS to try and shift substrate preference towards medium chain fatty acids. Seven mutations caused increased growth rates with hexanoate and octanoate, but not oleate. The mutations were of residues Val4{which corresponds to alignment index 69}, Trp5{70}, Tyr9{74}, Gln338{461}, Asp372{501}, His376{533}, Phe447{633} and Val451{637} (Table 2). These residues were not near the fatty acyl- or CoA-binding sites, but near the site of AMP exit. None of these indices were identified as group-specific positions in either LACS, MACS or SACS enzymes in this study.

### Group-specific residues in NRPSs

Eight residues had the highest Group Entropy scores in the non-ribosomal peptide synthetase (NRPS) group (Table 6). Complete GEnt results for NRPSs can be found in S4 Dataset. Group-specific residues identified in NRPSs by all methods used are summarized in S1 Dataset. Examination of residues was done with *Brevibacillus brevis* gramicidin synthetase phenylalanine-activating domain (sequence Brebregram, PDB ID: 1AMU). Phe234{373} forms part of the active site pocket near the α-phosphate of AMP and the carbonyl oxygen of the phenylalanine substrate. Gln432{643}, Glu441{652} and Glu443{654} form hydrogen bonds in NRPSs. Glu424{635} was found on a surface loop where it lies close to His344{533}. Tyr358{547}, Leu442{653} and Leu512{735} contribute to hydrophobic packing interactions within the enzyme. Tyr358{547} ring stacks with Phe402{609}. Of note is that none of the positions identified as critical to substrate preference in *B*. *brevis* gramicidin synthetase and *Paenibacillus* fusaricidin synthase were identified with high Group Entropy scores in NRPSs [77,78].

**Table 6:** Group entropy analysis of NRPSs.

Index	Residue Identity^a^	Group Entropy	Family Entropy	Highest Group Residue	Highest Family Residue	Conservation in Group	Residue Interactions^b^
643	Gln432	13.774	1.732	Gln	Met	98%	NE2 is 2.6Å from Gln414{625}.OE1
547	Tyr358	11.906	1.031	Tyr	Lys	70%	CB is 4.0Å from Phe402{609}.CE2 (ring stacking)
373	Phe234	10.080	4.158	Phe	His (65%)	74%	CE is 3.4Å from AMP.O3P CZ is 3.4Å from substrate Phe.O
653	Leu442	9.643	2.277	Leu	Pro	68%	CD2 is 4.0Å from Val460{672}.CG1
652	Glu441	9.386	1.249	Glu	Tyr	74%	OE2 is 2.9Å from Gln414{625}.NE2
735	Leu512	8.952	2.012	Leu	Lys	89%	CD1 is 3.9Å from Thr282{425}.OG1
635	Glu424	8.373	1.608	Glu	Trp	70%	OE2 is 3.2Å from His344{533}.NE2
654	Glu443	7.985	0.691	Gly	Ala	64% Gly 30% Glu	OE2 is 7.7Å from AMP.N3; OE2 is 6.7Å from AMP.O2’; OE2 is 2.8Å from Asn431{642}.ND2; OE2 is 3.0Å from Arg428{639}.N

^a^ Residue identity in Brebregram.

^b^ Structure PDB ID: 1AMU.

### Group-specific residues in MACSs

Ten residues were found to have the highest Group Entropy scores in the medium-chain fatty-acyl CoA synthetase (MACS) group (Table 7). Complete GEnt results for MACSs can be found in S5 Dataset. The group-specific residues identified in MACSs by all methods used are summarized in S1 Dataset. Examination of residues was done with human MACS (sequence Homsapacoa), by examining both the adenylation (PDB ID: 3DAY) and thioesterification (PDB IDs: 2WD9 & 3EQ6) conformations. Phe458{636} in the adenylation conformation makes hydrophobic contact with the adenine ring of the bound APC, an ATP analog. Several residues identified by GEnt interact with butyryl-CoA in the thioesterification conformation in structure 3EQ6. Tyr540{723} hydrogen bonds to the 3’ phosphate of the bound butyryl-CoA, while Arg501{680} forms a salt bridge to the β-5’ phosphate of the butyryl-CoA. Trp265{373} and Leu267{375} make hydrophobic contact with the bound butyryl-CoA. The bulky side chain of Trp265{373} constricts the active site channel to guide the CoA thiol group toward the fatty acid for thioesterification [5]. Leu267{375} lines the left pocket wall to allow ibuprofen to bind to MACS [5].

**Table 7:** Group entropy analysis of MACSs.

Index	Residue Identity^a^	Group Entropy	Family Entropy	Highest Group Residue	Highest Family Residue	Conservation in Group	Residue Interactions
168	Trp120	15.809	1.151	Trp	Trp	95%	CD1 is 4.0Å from Leu273{383}.CG (2WD9); CZ2 is 3.5Å from Val285{395}.CG2 (2WD9)
331	Met230	13.290	2.664	Met	Gly	94%	CE is 4.1Å from Met151{206}.CE (3DAY)
320	Tyr219	11.673	1.129	Tyr	Cys	82%	CE1 is 3.7Å from Ile266{374}.CA (2WD9); OH is 3.3Å from Ile269{378}.CG2 (2WD9)
373	Trp265	11.556	4.158	Trp	His (65%)	100%	CD1 is 4.0Å from BCO.N7 (3EQ6); CD1 is 4.4Å from BCO.C21 (3EQ6)
723	Tyr540	11.167	0.965	Tyr	Tyr	100%	OH is 2.7Å from BCO.O6 (3EQ6)
636	Phe458	10.221	2.190	Phe	Ile	100%	CE1 is 3.6Å from AMP.N3 (3EQ6)
185	Thr137	10.019	1.672	Thr	Asn	83%	OG1 is 2.6Å from Asp262{370}.OD2 (2WD9); CG2 is 4.0Å from Val554{737}.CG2 (3DAY)
680	Arg501	9.958	1.133	Arg	Trp	100%	NH2 is 2.8Å from BCO.O13 (3EQ6)
375	Leu267	9.938	0.949	Lys	Phe	86%	CB is 3.7Å from BCO.S1 (3EQ6)
654	Ser476	9.833	0.691	Phe	Ala	86%	OG is 2.9Å from Gly226{337].N (2WD9)

^a^ Residue identity in Homsapacoa.

Gly226{337} lies next to several residues that contact the bound APC molecule [5]. Ser476{654} hydrogen bonds with the main chain nitrogen of Gly226{337} to maintain enzyme folding. Thr137{185} provides a vital structural function in both conformations: during thioesterification the side chain hydroxyl of Thr137{185} provides an intradomain hydrogen bond with the side chain carboxylate of Asp262{370}, and an interdomain hydrophobic contact with Val554{737} in the adenylation conformation. Trp120{168}, Thr137{185}, Tyr219{320} and Met230{331} form hydrophobic contacts within MACSs.

### Group-specific residues in SACSs

Eight residues were found to have the highest Group Entropy scores in the short-chain fatty-acyl CoA synthetase (SACS) group (Table 8). Complete GEnt results for SACSs can be found in S6 Dataset. The group-specific residues identified in SACSs by all methods used are summarized in S1 Dataset. Examination of residues was done with *Salmonella enterica* acetyl-CoA synthetase (sequence Salentaco; PDB ID: 1PG3). Trp414{487} forms the pocket for the propyl group of the fatty acid substrate [10], which needs to be short for SACSs due to the presence of this large tryptophan residue. The conserved glycine at index 487 in MACSs and LACSs allows for a preference for longer fatty acid substrates [5]. Phe163{185} forms the active site pocket and is 3.3Å from the adenine ring of the bound CoA cofactor [10]. The hydroxyl of Thr438{538}, which has been reported to have abnormal angles, with ϕ = 70° and ψ = -118° [10], forms a hydrogen bond with the main chain nitrogen of Pro425{499}. Met141{163}, Thr278{336}, Trp395{465}, Leu477{591} and Trp598{729} participate in hydrophobic interactions within SACSs.

**Table 8:** Group entropy analysis of SACSs.

Index	Residue Identity^a^	Group Entropy	Family Entropy	Highest Group Residue	Highest Family Residue	Conservation in Group	Residue Interactions^b^
487	Trp414	25.885	3.534	Trp	Gly (93%)	100%	CE2 is 3.5Å from PRX.C3P
591	Leu477	16.768	4.207	Leu	Tyr (93%)	92%	CG is 3.8Å from Phe484{598}.CB; CD2 is 4.0Å from Val274{332}.CG2
465	Trp395	16.689	0.515	Trp	Trp	100%	CG is 3.7Å from Ala434{534}.CB; CZ3 is 3.6Å from His7{21}.CD2; CZ2 is 3.4Å from Val409{482}.CB
538	Thr438	15.284	3.312	Thr	Gly (93%)	100%	OG1 is 3.2Å from Thr424{498}.O; OG1 is 3.1Å from Pro425{499}.N
163	Met141	14.464	1.889	Met	Asn	100%	CG is 6.5Å from COA.N6A; CE is 3.9Å from Met329{395}.CG; CG is 3.4Å from Asp306{370}.OD2
185	Phe163	14.413	1.672	Phe	Asn	100%	CE1 is 3.3Å from COA.N6A
336	Thr278	13.913	3.206	Thr	His (73%)	100%	OG1 is 3.9Å from Leu259{316}.CB; CG2 is 4.4Å from Leu77{92}.CB
729	Trp598	13.627	2.162	Trp	Phe (65%)	100%	CD1 is 3.8Å from Pro571{696}.CB; NE1 is 3.9Å from Tyr576{705}.CD1

^a^ Residue identity in Salentaco.

^b^ Structure PDB ID: 1PG3.

### Group-specific residues in MMCSs

Eleven residues were found to have the highest Group Entropy scores in the methylmalonyl-CoA synthetase (MMCS) group (Table 9). Complete GEnt results for MMCSs can be found in S7 Dataset. The group-specific residues identified in MMCSs by all methods used are summarized in S1 Dataset. Examination of residues was done using *Rhodopseudomonas palustris* MMCS (sequence Rhopalmco; PDB IDs: 4FUT & 4FUQ). Several residues identified by GEnt contact substrates in the active site. The carbonyl oxygen of Arg299{485} hydrogen bonds with the adenine ring of ATP. The main chain carbonyl of the corresponding residue, Arg283{485}, of *Streptomyces coelicolor* MMCS (PDB ID: 3NYQ) also forms a hydrogen bond to the adenine ring of AMP. However, Arg283{485} also demonstrates a role in substrate binding through salt bridges to the bound methylmalonyl-coenzyme A (MCA) [79]. His209{375} in *R*. *palustris* MMCS hydrogen bonds to Ser277{457}. The equivalent residue in *S*. *coelicolor* MMCS, His189{375}, lines the active site pocket, even though the distance is too far (greater than 3.4Å, but within 3.8Å) to form hydrogen bonds to the methylmalonyl carbonyls of the bound MCA product. Ser277{457}, in addition to forming a hydrogen bond to His209{375}, makes hydrophobic contact with the adenine ring of ATP. The hydroxyl of the corresponding residue in *S*. *coelicolor* MMCS, Ser261{457}, also forms a hydrogen bond to the bound MCA [79]. Another residue that contacts MCA in *S*. *coelicolor* MMCS is Arg236{429}, which forms a salt bridge to the β-5’ phosphate of the bound MCA [79].

**Table 9:** Group entropy analysis of MMCSs.

Index	Residue Identity^a^	Group Entropy	Family Entropy	Highest Group Residue	Highest Family Residue	Conservation in Group	Residue Interactions^b^
738	Met486	13.578	1.446	Met	Ser	100%	CG is 6.0Å from ATP.O2A; SD is 4.0Å from Pro204{370}.CG; CE is 4.1Å from His209{375}.CB
485	Arg299	12.132	1.097	Arg	Gly	100%	O is 3.3Å from ATP.N6; NH1 is 3.3Å from His209{375}.ND1; Arg283{485}.NH2 is 2.9Å from MCA.OS4 (3NYQ); Arg283{485}.NH1 is 3.0Å from MCA.OS5 (3NYQ)
375	His209	11.148	0.949	His	Phe	100%	ND1 is 3.3Å from Arg299{485}.NH1; ND1 is 2.8Å from Ser277{457}.OG; His189{375}.CB is 3.4Å from MCA.OS4 (3NYQ); His189{375}.CB is 4.0Å from MCA.OS1 (3NYQ); His189{375}.CB is 4.5Å from of CS1.MCA (3NYQ)
594	Met364	10.855	1.218	Met	Asp	97%	SD is 4.1Å from Tyr361{591}.CB
457	Ser277	10.801	2.504	Ser	Gly	100%	C is 3.5Å from ATP.C8; OG is 2.8Å from His209{375}.ND1; Ser261{457}.OG is 2.6Å from MCA.OS4 (3NYQ)
421	Met247	10.071	0.882	Met	Cys	100%	CG is 3.8Å from Leu199{365}.CD1; SD is 4.2Å from Val213{379}.CG1; SD is 3.9Å from Ala214{381}.CB
576	Glu351	9.747	1.318	Glu	Cys	90%	OE2 is 2.6Å from Arg373{605}.NE
465	His285	9.657	0.515	His	Trp	90%	ND1 is 3.7Å from Pro319{534}.CG (ring stacking); NE2 is 2.8Å from Val296{482}.O; His269{465}.NE2 is 2.9Å from Glu282{484}.OE1 (3NYQ)
453	Phe273	9.547	1.151	Phe	Cys	100%	CE2 is 3.7Å from His294{474}.NE2; CE1 is 3.7Å from Met240{413}.CE; CD1 is 3.9Å from Leu246{420}.CG
413	Met240	9.541	0.695	Met	Gln	84%	CE is 3.7Å from Phe273{453}.CE1
429	Arg255	9.539	0.657	Arg	Met	100%	Arg236{429}.NH2 is 2.7Å from MCA.O22 (3NYQ)

^a^ Residue identity in Rhopalmco.

^b^ Measurements from 4FUT structure, unless otherwise noted.

Several more group-specific residues from hydrogen bonds and salt bridges. The side chain carboxylate of Glu351{576} forms a salt bridge on the surface of the molecule with Arg373{605}. His285{465} ring stacks with Pro319{534} and forms a hydrogen bond with the carbonyl oxygen of Val296{482}. The side chain of the corresponding residue of *S*. *coelicolor* MMCS, His269{465}, forms a salt bridge with the side chain carboxylate of Glu282{484}, which is 3.7Å from the adenosine amino group of the bound AMP [79]. Interestingly, the equivalent glutamate in *R*. *palustris* MMCS, Glu298{484}, is too distant from His285{465} to form a salt bridge, but does form a hydrogen bond to the adenine amino group of the bound ATP [20]. Met240{413}, Met247{421}, Phe273{453}, Met364{594}, and Met486{738} all form hydrophobic contacts in MMCSs. Met486{738} functions to form the binding pocket wall, at a distance of 6Å from an oxygen on the α-phosphate of the bound ATP.

### Group-specific residues in ACLs

Eight residues were found to have the highest Group Entropy scores in the aryl-CoA ligase (ACLs) group (Table 10). Complete GEnt results for ACLs can be found in S8 Dataset. The group-specific residues identified in ACLs by all methods used are summarized in S1 Dataset. Examination of residues was done with *Alcaligenes* 4-chlorobenzoyl:CoA ligase (CBL, sequence Alcalc4b; PDB ID: 3CW9). Two residues identified by GEnt interact with the substrates. Asn411{650} hydrogen bonds to the α-phosphate of AMP, but only in the thioesterification conformation as the pyrophosphate of ATP blocks Asn411{650} from entering the site [22]. His207{373}, which hydrogen bonds to Glu410{649}, also interacts with the 4-chlorobenzoate carboxylate during the adenylation reaction [22] and the acid anhydride bond that joins AMP and 4-chlorobenzoate [21]. Mutation of His207{373} resulted in a significant decrease in activity and catalytic efficiency with 4-chlorobenzoate [22] (Table 2).

**Table 10:** Group entropy analysis of ACLs.

Index	Residue Identity^a^	Group Entropy	Family Entropy	Highest Group Residue	Highest Family Residue	Conservation in Group	Residue Interactions^b^
650	Asn411	7.433	2.197	Asn	Trp	100%	ND2 is 3.0Å from AMP.O1P
713	Cys465	6.573	1.291	Cys	Val	55%	SG is 4.7Å from Val240{412}.CG1; SG is 3.7Å from Tyr479{727}.OH
186	Pro86	4.740	0.764	Trp	Pro	29% Trp	CG is 4.4Å from Pro62{162}.CD; CB is 3.8Å from Val109{227}.CG1
134	Leu35	4.544	1.390	Trp	Leu	41% Trp 41% Leu	CD1 is 3.7Å from Ile69{169}.CG1; CD1 is 3.8Å from Leu21{115}.CD2
373	His207	4.196	4.158	Gln	His	100%	NE2 is 2.8Å from Glu410{649}.OE2; NE2 is 4.4Å from 01A.O5P
661	Gly422	4.139	0.704	Tyr	Met	29% Tyr	CA is 3.9Å from Val430{670}.CG1; CA is 4.4Å from Val427{667}.CG1
654	Ser415	4.027	0.691	Arg	Ala	49% Arg	OG is 3.3Å from Thr164{325}.O
643	Met404	3.951	1.732	Met	Met	84%	CE is 4.0Å from Gly163{324}.C; SD is 7.4Å from AMP.O3P; SD is 3.5Å from His413{652}.N

^a^ Residue identity in Alcalc4b.

^b^ Structure PDB ID: 3CW9.

The hydroxyl of Ser415{654} lies near the main chain carbonyl oxygen of Thr164{325}. Leu35{134}, Pro86{186}, Met404{643}, Gly422{661} and Cys465{713} make hydrophobic contacts within ACLs. Pro86{186} lies right before Arg87{187}, which interacts with the α-phosphate of the bound 4-chlorophenacyl-CoA molecule. Thus, Pro86{186} likely helps to position Arg87{187} for proper contact with the substrate [22].

### Group-specific residues in FAALs

Nine residues were found to have the highest Group Entropy scores in the fatty acid-AMP ligase (FAAL) group (Table 11). Complete GEnt results for FAALs can be found in S9 Dataset. The group-specific residues identified in FAALs by all methods used are summarized in S1 Dataset. Examination of residues was done with *E*. *coli* fatty acid-AMP ligase (sequence Ecolifaal; PDB ID: 3PBK). An important note is that each position is three numbers higher in the PDB structure than in our sequence alignment. Position numbers from the PDB structure are used here. None of the residues identified by GEnt in FAALs interact with the substrate. One residue, Pro540{729}, forms a hydrogen bond between its carbonyl oxygen and the hydroxyl of Ser543{732}. Arg469{649} forms a salt bridge with Glu366{516}, which is in the insertion motif in FAALs. This blocks the binding of CoA, allowing for only the adenylation reaction to occur, rather than additional acyl-CoA synthetase activity [37]. The remainder of the residues that scored highly for Group Entropy (Trp224{368}, Leu245{390}, Trp262{408}, Phe279{425}, Cys284{430}, Phe494{675} and Ala557{746}) are involved in hydrophobic packing within FAALs. Three residues, Trp224{368}, Leu245{390} and Phe279{425}, appear to line the active site pocket, but are more than 5Å from the bound dodecacyl-adenylate molecule. Leu245{390}, which lies at a position that is a 78% conserved glycine within the entire alignment, is 7.5Å from the C_ω_ of the bound dodecacyl-adenylate molecule. A glycine at this position could allow enzymes in other families to accommodate longer fatty acid chains.

**Table 11:** Group entropy analysis of FAALs.

Index	Residue Identity^a^	Group Entropy	Family Entropy	Highest Group Residue	Highest Family Residue	Conservation in Group	Residue Interactions^b^
368	Trp224	17.034	0.993	Trp	Val	100%	NE1 is 3.3Å from Phe255{401}.CE1 (ring stacking); NE1 is 3.6Å from Trp262{408}.CE3
408	Trp262	16.450	1.188	Trp	Phe	100%	CE3 is 3.6Å from Trp224{368}.NE1; CB is 3.6Å from Phe255{401}.CE1
746	Ala557	14.967	2.349	Cys	Leu (79%)	59% Cys	CB is 3.9Å from Phe494{675}.CE1; CB is 4.3Å from Ile492{673}.CG2
425	Phe279	12.632	1.870	Phe	Thr (62%)	94%	CZ is 4.1Å from Trp224{368}.CH2; CE2 is 10.4Å from 1ZZ.O1
430	Cys284	11.704	1.815	Cys	Leu (66%)	82%	SG is 3.7Å from Trp262{408}.CE3; SG is 4.5Å from Leu263{409}.CG; CB is 4.4Å from Phe323{472}.CZ
729	Pro540	11.188	2.162	Pro	Phe (65%)	71%	O is 2.9Å from Ser543{732}.OG
390	Leu245	11.000	2.126	Phe	Gly (78%)	35% Phe 41% Leu	CD1 is 7.5Å from OZZ.C1; CD1 is 4.0Å from Leu215{358}.CD1; CD1 is 4.7Å from Cys221{365}.SG
649	Arg469	10.489	1.876	Arg	Tyr	59%	NH2 is 2.8Å from Glu366{516}.OE1; NH2 is 3.2Å from Phe530{719}.O
675	Phe494	10.094	0.823	Phe	Val	100%	CE2 is 4.2Å from Ile502{688}.CD1; CD2 is 3.6Å from Lys558{747}.CB

^a^ Residue identity in Ecolifaal.

^b^ Structure PDB ID: 3PBK.

An additional note is that the activity of the Fad32 protein from mycobacteria, an FAAL involved in the synthesis of mycolic acids, is decreased by phosphorylation on Thr552, which is on an accessible loop [80]. However, structural alignment (not shown) of *E*. *coli* FAAL (PDB ID: 3PBK) with Fad32 from *M*. *tuberculosis* (PDB ID: 5HM3) revealed that Thr552 in Fad32 is in an insertion motif which is an extended loop not found in other aligned FAALs, and thus has no equivalent index position in our alignment. This suggests that this phosphorylation might be unique to mycobacteria.

### Group-specific residues in FadD10s

Ten residues were found to have the highest Group Entropy scores in the mycobacterial FadD10 long chain fatty acyl-CoA ligase (FadD10) group (Table 12). Complete GEnt results for FadD10s can be found in S10 Dataset. The group-specific residues identified in FadD10s by all methods used are summarized in S1 Dataset. Examination of residues was done with *Mycobacterium tuberculosis* FadD10 (sequence Myctubfd10; PDB ID: 4IR7). Similar to the FAALs, the residue position number in Myctubfd10 in our alignment is one higher than that of the position in the structural coordinates, which are the position numbers reported here. Only one residue identified by GEnt interacts with the substrate in FadD10s. Trp231{381} lies 3.7Å from the C_ω_ of the bound dodecacyl-adenylate substrate [81]. Therefore, Trp231{381} may influence the length of the fatty acid substrate that the enzyme could bind. Although not identified as group specific in Luciferases, a T251S mutation at index 381 improved luminescence with aminoluciferins [82] (Table 2); this change in substrate preference coincides with the residue’s important location in the substrate-binding pocket.

**Table 12:** Group entropy analysis of FadD10s.

Index	Residue Identity^a^	Group Entropy	Family Entropy	Highest Group Residue	Highest Family Residue	Conservation in Group	Residue Interactions^b^
381	Trp231	13.801	0.325	Trp	Tyr	100%	CE3 is 3.7Å from OZZ.C1
467	Phe305	11.010	0.651	Phe	Trp	100%	CD2 is 3.6Å from Val275{431}.CG1
395	Gly245	10.229	1.029	Gly	Met	100%	CA is 4.0Å from Gly76{164}.CA; O is 2.9Å from Cys36{118}.N (4ISB)
620	Val404	10.172	2.303	Val	Tyr	100%	CG1 is 3.8Å from Glu321{490}.CB; CG1 is 3.8Å from Thr322{491}.CG2; CG2 is 3.3Å from Asn384{587}.ND2
673	Tyr456	9.986	1.476	Tyr	Ile	86%	OH is 3.4Å from Ala529{750}.CB; CD2 is 3.9Å from Ala526{747}.CB; CD1 is 4.2Å from Leu525{746}.CD1; OH is 4.2Å from Val507{728}.CG2
641	Ser425	9.929	2.621	Ser	Lys	100%	OG is 3.1Å from Glu457{674}.OE1
587	Asn384	9.846	1.172	Asn	Ile	100%	ND2 is 3.0Å from Ile379{577}.O
548	Tyr354	9.641	1.739	His	Ile	57% His 43% Tyr	OH is 2.9Å from Gly369{564}.O; CB is 3.7Å is Trp403{619}.CZ3; CE2 is 3.8Å from Pro363{560}.CD
464	Asp302	9.564	0.951	Asp	Val	100%	OD2 is 3.3Å from Ser272{428}.OG
672	Cys455	9.564	2.456	Cys	Val (78%)	100%	CB is 3.9Å from Val440{656}.CG1; N is 3.0Å from Asp441{657}.OD1; SG is 3.5Å from Pro437{653}.CA

^a^ Residue identity in Myctubfd10.

^b^ Measurements from 4IR7 structure, unless otherwise noted.

Five other residues from hydrogen bonds within FadD10s. The apoenzyme structure (PDB ID: 4ISB) showed a hydrogen bond between the main chain nitrogen of Cys36{118} and the carbonyl oxygen of Gly245{395}. Ser425{641}, which lies in the linker motif connecting the amino-terminal and carboxy-terminal domains [81], forms a hydrogen bond to the side chain carboxylate of Glu457{674}. Asn384{587} and Tyr354{548} both maintain loop structures by forming hydrogen bonds with the main chain carbonyl oxygens of Ile379{577} and Gly369{564}, respectively. The side chain carboxylate of Asp302{464} hydrogen bonds with the hydroxyl of Ser272{428}. The remainder of the residues with the highest Group Entropy scores (Gly245{395}, Phe305{467}, Tyr354{548}, Val404{620}, Cys455{672} and Tyr456{673}) are involved in hydrophobic packing within FadD10s.

### Common group-specific positions

Residue positions with high Group Entropy scores in multiple groups would represent critical sites of evolutionary differences. There were eleven index positions identified by GEnt in multiple groups. Five common group-specific index positions line the active site pocket, including indices 185, 320, 373, 375 and 650. Index 650 had the highest Group Entropy score in three groups: Luciferases, LACSs and ACLs. The residue at this index appears to hydrogen bond to the α-phosphate of the AMP, but in a conformation dependent manner. The side chain of Trp444{650} in LACSs hydrogen bonds to the α-phosphate of the AMP moiety [3]. In CBL, an ACL, the side chain of Asn411{650} hydrogen bonds to the α-phosphate of the AMP when the enzyme is in the thioesterification conformation only [22]. In the *L*. *cruciata* luciferase Gln450{650} was on a surface loop, removed from the active site. It is possible that this structure was in the adenylate-forming conformation, as luciferases do not carry out a thioesterification reaction. The residue at this index position throughout the entire alignment tends to be polar, being asparagine in ACLs, MMCSs, FAALs and FadD10s and arginine in SACSs, MACSs and NRPSs. Although index 650 was the position with the 54^th^ highest Group Entropy score in MACSs, Arg472{650} in human MACS (sequence Homsapacoa) was examined for differences in both adenylation and thioesterification conformations, as structures were available for both. In the thioesterification conformation (PDB ID: 2WD9) the side chain of Arg472{650} was 2.8Å from the bound ibuprofen and formed a hydrogen bond (3.1Å) from the side chain hydroxyl of the conserved Thr221{322}. Also seen in the thioesterification conformation is a conserved interdomain salt bridge between Arg472{650} and Glu365{490}, which serves to block further ATP binding [5]. In the adenylation conformation of human MACS (PDB ID: 3DAY) a new interdomain salt bridge is formed between Arg472{650} and Glu407{572}, which lies right beside the invariant Gly408{573}.

Index 373 was identified by GEnt in NRPSs, MACSs and ACLs. Histidine is 65% conserved in the entire alignment at index 373. In NRPSs Phe234{373} forms part of the active site pocket near the α-phosphate. In CBL His207{373} binds to the acid anhydride bond that connects the AMP and 4-chlorobenzoate moieties [21]. As inferred by studying a H207A mutant, the side chain of His207{373} also interacts with the 4-chlorobenzoate during the first part of the reaction [22] (Table 2). In human MACS Trp265{373} acts to narrow the pantetheine channel in the thioesterification conformation, which in turn directs the thiol of the CoA substrate to the correct position for nucleophilic attack on the fatty acyl-adenylate intermediate [5]. Thus, the residue at index 373 lies near the actual site of adenylate bond formation during catalysis.

Index 320 was identified by GEnt in LACSs and MACSs, and was also identified by the majority of other methods used to determine group-specific residues in ACLs and FAALs (S1 Dataset). In the entire alignment the residue at index 320 tends to be aliphatic. In the groups noted the residue at index 320 is involved in hydrophobic packing. In ACLs Phe159{320} contributes to hydrophobic packing. Ala182{320} in *T*. *thermophilus* LACS is 5.7Å from the bound ANP and hydrophobically contacts Tyr196{334}, also identified by GEnt in LACSs. Tyr219{320} in human MACS contacts Ile266{374}, which forms the left pocket wall in the active site [5]. In FAALs Gln182{320} is nearly 7Å from the bound dodecanoyl-AMP. Hence, this position contributes to the active site shape.

Index 185, identified by GEnt in MACSs and SACSs, has enzyme-specific functions. In *S*. *enterica* SACS Phe163{185} hydrophobically contacts the adenine ring of the bound CoA cofactor in the active site pocket [10]. In human MACS the hydroxyl of Thr137{185} forms an intradomain hydrogen bond with the side chain carboxylate of Asp262{370} during thioesterification, but makes interdomain hydrophobic contact with Val554{737} in the adenylation conformation [5]. Luciferases, ACLs, LACSs and FadD10s tend to have an asparagine at this index position.

Index 375, identified by GEnt in MACSs and MMCSs, lines the hydrophobic pocket wall of the active site where substrates bind in both groups. In human MACS Leu267{375} lines the left pocket wall and also contacts the butyryl-CoA near the sulfur atom in the thioesterification conformation [5]. In *S*. *coelicolor* MMCS His189{375} lines the active site pocket and contacts of the bound MCA product [79]. His209{375} in *R*. *palustris* MMCS hydrogen bonds with Ser277{457} and also makes hydrophobic contact with Met486{738} and Arg299{485}, all of which were also identified by GEnt. These three residues contacted by His209{375} all play important roles in MMCSs (noted above). Although not identified as group specific in Luciferases, a F247S mutant at index 375 in *Photinus pyralis* luciferase increased light output with aminoluciferin, but with a high K_m_ value [82] (Table 2), indicating that it lies close to the substrate.

The residue at index 654, identified by GEnt in ACLs, MACSs, and NRPSs, forms bonds to maintain the structure of these enzymes. Though MACSs mostly have a phenylalanine at index 654, Ser476{654} in human MACS hydrogen bonds with the main chain nitrogen of Gly226{337}, which lies next to several residues that contact the bound APC molecule [5]. The hydroxyl of Ser415{654} in CBL hydrogen bonds to the main chain carbonyl oxygen of Thr164{325}. In *B*. *brevis* gramicidin synthetase, a NRPS, Glu443{654} hydrogen bonds to the main chain nitrogen of the invariant Arg428{639} and the side chain of Asn431{642}.

Five additional common group-specific index positions, indices 465, 643, 652, 721 and 729, are involved in hydrophobic interactions within most enzymes. Index 721 in the carboxyl-terminal domain had high Group Entropy scores in both Luciferases and LACSs. In most groups, the residue at index 721 tends to be a hydrophobic residue. In Luciferases Lys512{721} forms a salt bridge with the side chain carboxylate of Glu455{655} on the enzyme surface. In LACSs Trp505{721} contributes to hydrophobic packing. As index 721 lies in the carboxy-terminal domain, it is possible that the binding contacts for this residue might also change upon a shift in domain alternation. In human MACS the C_α_ of Tyr538{721} is 3.6Å from the O4 position of the bound butyryl-CoA in the 3EQ6 structure [5]. Thus, it may also play a role in coenzyme A binding.

Index 652 was identified by GEnt in NRPSs and Luciferases, and was also identified by the majority of other methods used to determine group-specific residues in ACLs, FAALs and MACSs (S1 Dataset). The residue at index 652 appears important to maintain enzyme structure, though through different mechanisms depending upon the enzyme. Index 652 is in a turn in the enzyme structure. Pro452{652} in Luciferases may be important for the structure of the loop containing Gln450{650}, also identified by GEnt in Luciferases. Proline at this position is unique to Luciferases. In MACS Gly474{652} also contributes to the structure of this turn. However, in NRPSs and ACLs the residue at index 652 forms a hydrogen bond. In NRPSs Glu441{652} forms a hydrogen bond to Gln414{625}. In ACLs the carbonyl oxygen of the conserved Thr164{325} forms a hydrogen bond to His413{652} during the thioesterification conformation [21]. In FAALs Trp472{652} is involved in hydrophobic packing.

Index 643 was identified by GEnt in NRPSs and ACLs. In the entire alignment, the residue at index 643 also tends to be aliphatic. In CBL Met404{643} contributes to hydrophobic packing. In gramicidin synthetase NRPS Gln432{643} hydrogen bonds with Gln414{625}, which is also contacted by Glu441{652} noted above. These interactions appear to be unique to NRPSs.

Index 465 was identified by GEnt in SACSs and MMCSs. In *S*. *enterica* SACS Trp395{465} contributes to hydrophobic packing. The side chain of His269{465} in *S*. *coelicolor* MMCS forms a salt bridge to Glu282{484}, which is close to the amino group of the bound AMP adenosine [79]. In both groups the residue at index 465 makes hydrophobic contact with the residue at index 534 and also contacts the residue at index 482.

Index 729, which is a 65% conserved phenylalanine in the entire alignment, was identified by GEnt in SACSs and FAALs. In *E*. *coli* FAAL Pro540{729} contributes to the structure of a surface loop and forms a hydrogen bond to Ser543{732}. In *S*. *enterica* SACS Trp598{729} is involved in hydrophobic packing.

## Discussion

This project aligned a total of 374 amino acid sequences of class I adenylate-forming enzymes. Five residue positions were invariant, with 22 additional residues conserved in at least 80% of all of the aligned sequences, and 56 more residues conserved in at least 60%. Many of these residues have been studied by site-directed mutagenesis in several groups (Table 2). A threonine at index 322 and glutamate at index 490 coordinate the Mg^2+^ ion. Several highly conserved residues coordinate the AMP/ATP molecule, including indices 323, 457, 486, 489, 624, 639 and 740. Thirteen conserved positions, including indices 142, 145, 157, 325, 326, 329, 424, 456, 487, 573, 632, 686 and 734, contribute to hydrophobic packing within the enzyme. Five conserved residues at indices 321, 330, 591, 655 and 657 form hydrogen bonds or salt bridges that maintain enzyme folding. Four conserved residues at indices 465, 486, 487 and 489 line in the fatty acid-binding pocket of *T*. *thermophilus* LACS. A high proportion of the conserved residues were glycines, a phenomenon seen in several other enzyme families [38, 65–68]. These conserved residues are responsible for structural and functional aspects common to all superfamily members, such as magnesium and ATP binding, and hydrophobic packing.

Ten highly conserved sequence motifs were identified, half of which had been previously identified in the adenylation domain of NRPSs [70]. Motifs 1, 2, 3, 4, 7, 9 and 10 line the active site of *T*. *thermophilus* LACS. Motif 1 encompasses the linker (L) motif that connects the two domains. Motif 3 includes the P-loop in the phosphate-binding site. The adenine (A) motif that interacts with the adenine of AMP was not found in the ten motifs identified. Most sequence hits from a MAST search of a protein database using the motifs were adenylate-forming enzymes, including D-alanine-D-alanyl carrier protein ligase which was not included in this project. Two enzymes also identified by the MAST search were cinnamyl alcohol dehydrogenase and phenylalanine racemase, but they did not show functional similarities to adenylate-forming enzymes.

Phylogenetic analysis verified nine distinct groups of class I adenylate-forming enzymes, which were then used to identify group-specific residues. Surprisingly, all of the ACSs (SACSs, MACSs and LACSs) were not on adjacent clades, with LACSs being more related to Luciferases than the other ACSs. FAALs and NRPSs are located on neighboring clades. Both groups attach the reaction intermediate to a carrier protein, rather than CoA.

Group entropy analysis, as well as other methods, were employed to determine the residues unique to each group. Unlike the residue positions conserved in the entire alignment, these group-specific positions are responsible for unique structural interactions or functional differences in each group. Eleven index positions identified by GEnt in multiple groups represent important sites of evolutionary differences. These common index positions include indices 185, 320, 373, 375 and 465 from the amino-terminal domain, index 643 from the linker motif, and indices 650, 652, 654, 721 and 729 from the carboxyl-terminal domain. Five common group-specific index positions line the active site pocket, including indices 185, 320, 373, 375 and 650. The residue at index 650 interacts with the α-phosphate of AMP [3,22], while the residue at index 373 lies where the acid anhydride bond between AMP and the substrate occurs [5,21,22]. Index 320 contributes to the shape the active site pocket [5]. The residue at index 185 interacts with coenzyme A [10], while the residue at index 375 interacts with the CoA-bound product [5,79]. Index 721 also contacts the butyryl-CoA in human MACS [5]. These positions are likely responsible for differences in catalytic function or substrate preference.

**Fig 7:**
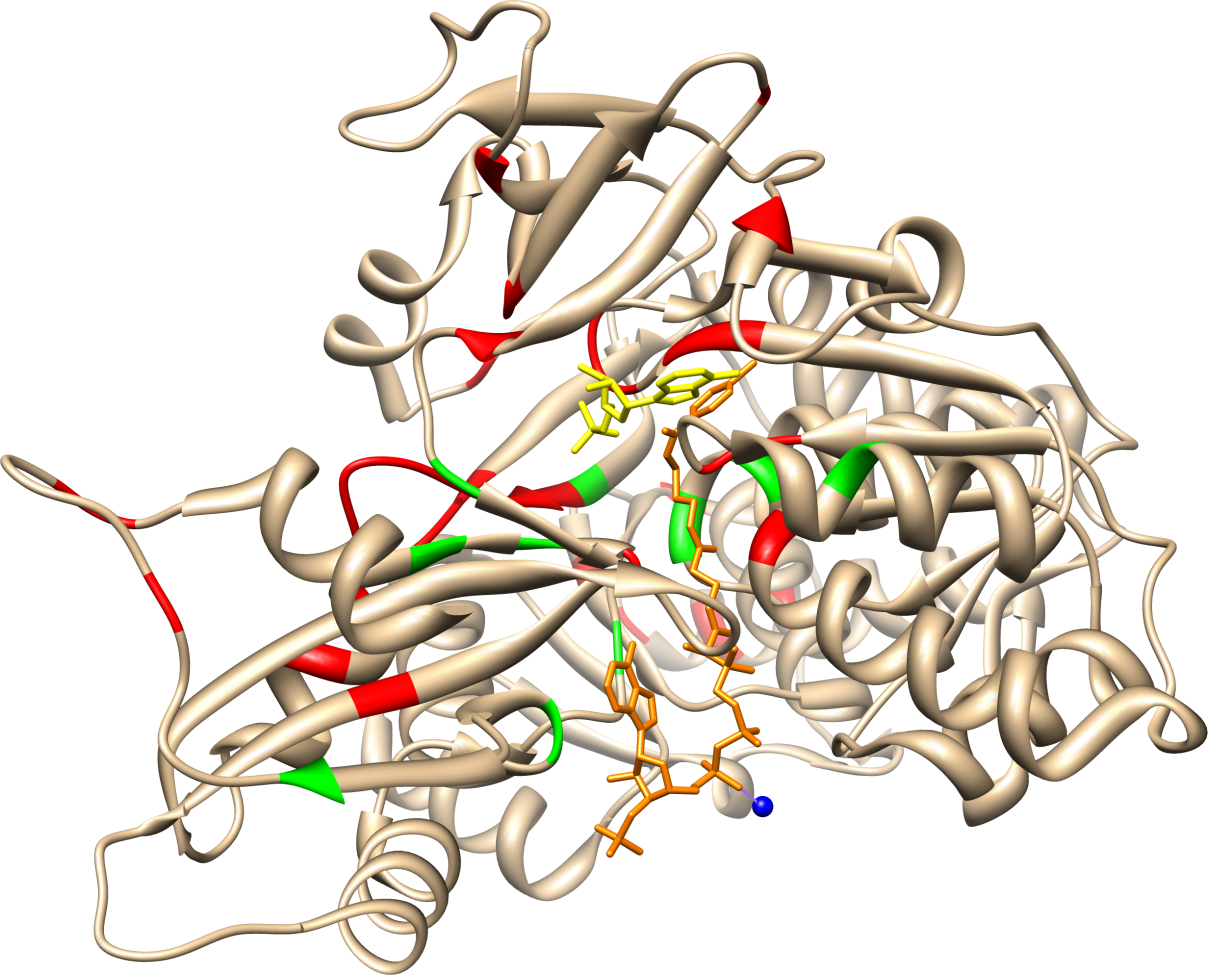
Conservations in 4-Chlorobenzoyl:CoA ligase from *Alcaligenes* (PDB ID: 3CW9). Residues conserved throughout the entire superfamily are highlighted red and the eleven common group-specific positions are highlighted green. Also shown is 4-chlorobenzoyl-CoA in orange, AMP in yellow and Mg^2+^ in blue. While the AMP is surrounded by more overall conserved residues (red), the 4-chlorobenzoyl-CoA molecule is surrounded by more group-specific conservations (green).

The residue at index 654 forms group-specific hydrogen bonds. Six common group-specific index positions, indices 320, 465, 643, 652, 721 and 729, are involved in hydrophobic interactions within most enzymes. In addition, four of these six positions (465, 643, 652 and 721) also participate in unique hydrogen bonds or salt bridges in specific families. These positions are critical for the unique structural differences in each enzyme group. While most of the residues conserved throughout the entire superfamily are found throughout the structure and specifically near the bound AMP, which is utilized by all members of the superfamily, several of the common group-specific residues lie closer to the substrate and coenzyme A molecules (Fig 7).

Additionally, there are three index positions identified by GEnt in specific groups, not common to multiple groups, that might influence the length of the fatty acid substrate. A glycine is conserved at index 487 in all groups aligned except SACSs. In SACSs a large tryptophan at index 487 necessitates a smaller fatty acid chain to bind [10], while in MACSs and LACSs a glycine at index 487 allows for longer chain fatty acids to bind [5]. Second, index 390 is a 78% conserved glycine within the entire alignment. However, in FAALs the residue at index 390 is a leucine that is 7.5Å from the C_ω_ of the bound dodecanoyl-AMP molecule, possibly restricting the length of the fatty acid in this group. Lastly, a tryptophan at index 381 is 3.7Å from the C_ω_ of the bound dodecanoyl-AMP substrate in FadD10s [81]. The amino acid composition at index 381, however, is variable in the different groups aligned. The group-specific conservations identified here, as well as the positions conserved in the entire superfamily, could serve as interesting targets for site-directed mutagenesis by other researchers.

## Supporting information

S1 File Complete alignment of 374 class 1 adenylate-forming enzyme sequences (MSF format). (MSF)

S1 Fig Unrooted bootstrapped parsimony tree of class 1 adenylate-forming enzymes. Branches are color-coded based on enzyme type: green = luciferases, purple = LACS, cyan = ACL, blue = MMCS, pink = FAAL, orange = NRPS, yellow = FadD10, navy = SACS and red = MACS.
(TIF)

S1 Dataset Results from all methods used to determine group-specific conservations for every group. (XLSX)

S2 Dataset Complete GEnt results of Luciferases. (TXT)

S3 Dataset Complete GEnt results of LACSs. (TXT)

S4 Dataset Complete GEnt results of NRPSs. (TXT)

S5 Dataset Complete GEnt results of MACSs. (TXT)

S6 Dataset Complete GEnt results of SACSs. (TXT)

S7 Dataset Complete GEnt results of MMCSs. (TXT)

S8 Dataset Complete GEnt results of ACLs. (TXT)

S9 Dataset Complete GEnt results of FAALs. (TXT)

S10 Dataset Complete GEnt results of FadD10s. (TXT)

## References

[1] Gulick AM. Conformational dynamics in the acyl-CoA synthetases, adenylation domains of non-ribosomal peptide synthetases, and firefly luciferases. ACS Chem Biol. 2009; 4: 811–827. 10.1021/cb900156h 19610673 PMC2769252

[2] Schmelz S, Naismith JH. Adenylate Forming Enzymes. Curr Opin Struct Biol. 2009; 19: 666–671. 10.1016/j.sbi.2009.09.004 19836944 PMC3313645

[3] Hisanaga Y, Ago H, Nakagawa N, Hamada K, Ida K, Yamamoto M, et al Structural basis of the substrate-specific two-step catalysis of long chain fatty acyl CoA synthetase dimer. J Biol Chem. 2004; 279: 31717–31726. 10.1074/jbc.M400100200 15145952

[4] Chang K, Xiang H, Dunaway-Mariano D. Acyl-Adenylate Motif of the Acyl-Adenylate/Thioester-Forming Enzyme Superfamily: A Site-Directed Mutagenesis Study with the *Pseudomonas* sp. Strain CBS3 4-Chlorobenzoate: Coenzyme A Ligase. Biochemistry 1997; 36: 15650–15659. 10.1021/bi971262p 9398293

[5] Kochan G, Pilka ES, vonDelft F, Oppermann U, Yue WW. Structural snapshots for the conformation-dependent catalysis of human medium-chain acyl-coenzyme A synthetase ACSM2A. J Mol Biol. 2009; 388: 997–1008. 10.1016/j.jmb.2009.03.064 19345228

[6] Conti E, Franks NP, Brick P. Crystal structure of firefly luciferase throws light on a superfamily of adenylate-forming enzymes. Structure. 1996; 4: 287–298. 8805533 10.1016/s0969-2126(96)00033-0

[7] Conti E, Stachelhaus T, Marahiel MA, Brick P. Structural basis for the activation of phenylalanine in the non-ribosomal biosynthesis of gramicidin S. EMBO J. 1997; 16: 4174–4183. 9250661 10.1093/emboj/16.14.4174 PMC1170043

[8] Airas RK. Magnesium dependence of the measured equilibrium constants of aminoacyl-tRNA synthetases. Biophys Chem. 2007; 131: 29–35. 10.1016/j.bpc.2007.08.006 17889423

[9] Yonus H, Neumann P, Zimmerman S, May JJ, Marahiel MA, Stubbs MT. Crystal structure of DltA. Implications for the reaction mechanism of non-ribosomal peptide synthetase adenylation domains. J Biol Chem. 2008; 283: 32484–32491. 10.1074/jbc.M800557200 18784082

[10] Gulick AM, Starai VJ, Horswill AR, Homick KM, Escalante-Semerena JC. The 1.75 Å crystal structure of acetyl-CoA synthetase bound to adenosine-5’-propylphosphate and coenzyme A. Biochemistry. 2003; 42: 2866–2873. 10.1021/bi0271603 12627952

[11] Glick BS, Rothman JE. Possible role for fatty acyl-coenzyme A in intracellular protein transport. Nature. 1987; 326: 309–312. 10.1038/326309a0 3821906

[12] Li ZN, Hongo S, Sugawara K, Sugahara K, Tsuchiya E, Matsuzaki Y, et al The sites for fatty acylation, phosphorylation and intermolecular disulphide bond formation of influenza C virus CM2 protein. J Gen Virol. 2001; 82: 1085–1093. 10.1099/0022-1317-82-5-1085 11297683

[13] Murakami K, Ide T, Nakazawa T, Okazaki T, Mochizuki T, Kadowaki T. Fatty-acyl-CoA thioesters inhibit recruitment of steroid receptor co-activator 1 to alpha and gamma isoforms of peroxisome-proliferator-activated receptors by competing with agonists. Biochem J. 2001; 353: 231–238. 11139385 10.1042/0264-6021:3530231 PMC1221563

[14] Tejima K, Ishiai M, Murayama SO, Iwatani S, Kajiwara S. *Candida albicans* fatty acyl-CoA synthetase, CaFaa4p, is involved in the uptake of exogenous long-chain fatty acids and cell activity in the biofilm. Curr Genet. 2017 10.1007/s00294-017-0751-2 28942495

[15] Banchio C, Gramajo H. A stationary-phase acyl-coenzyme A synthetase of *Streptomyces coelicolor* A3(2) is necessary for the normal onset of antibiotic production. Appl Environ Microbiol. 2002; 68(9): 4240–4246. 10.1128/AEM.68.9.4240-4246.2002 12200271 PMC124087

[16] Ray S, Chatterjee E, Chatterjee A, Paul K, Chowdhury R. A *fadD* mutant of *Vibrio cholerae* is impaired in the production of virulence factors and membrane localization of the virulence regulatory protein TcpP. Infect Immun. 2011; 79: 258–266. 10.1128/IAI.00663-10 21041490 PMC3019873

[17] Lucas RL, Lostroh CP, DiRusso CC, Spector MP, Wanner BL, Lee CA. Multiple factors independently regulate hilA and invasion gene expression in *Salmonella enterica* serovar typhimurium. J Bacteriol. 2000; 182: 1872–1882. 10.1128/JB.182.7.1872–1882.2000 10714991 PMC101869

[18] Dunphy KY, Senaratne RH, Masuzawa M, Kendall LV, Riley LW. Attenuation of *Mycobacterium tuberculosis* functionally disrupted in a fatty acyl-coenzyme A synthetase gene *fadD5*. J Infect Dis. 2010; 201(8): 1232–1239. 10.1086/651452 20214478 PMC3225055

[19] Feng S, Xu C, Yang K, Wang H, Fan H, Liao M. Either fadD1 or fadD2, Which Encode acyl-CoA Synthetase, Is Essential for the Survival of *Haemophilus parasuis* SC096. Front Cell Infect Microbiol. 2017; 7: 72 10.3389/fcimb.2017.00072 28361037 PMC5350145

[20] Crosby HA, Rank KC, Rayment I, Escalante-Semerena JC. Structure-guided expansion of the substrate range of methylmalonyl coenzyme A synthetase (MatB) of *Rhodopseudomonas palustris*. Appl Environ Microbiol. 2012; 78(18): 6619–6629. 10.1128/AEM.01733-12 22773649 PMC3426712

[21] Reger AS, Wu R, Dunaway-Mariano D, Gulick AM. Structural characterization of a 140 degrees domain movement in the two-step reaction catalyzed by 4-chlorobenzoate:CoA ligase. Biochemistry. 2008; 47(31): 8016–8025. 10.1021/bi800696y 18620418 PMC2666193

[22] Wu R, Cao J, Lu X, Reger AS, Gulick AM, Dunaway-Mariano D. Mechanism of 4-chlorobenzoate:coenzyme a ligase catalysis. Biochemistry. 2008; 47(31): 8026–8039. 10.1021/bi800698m 18620421 PMC3694354

[23] Ferrer JL, Austin MB, Stewart C, Noel JP. Structure and function of enzymes involved in the biosynthesis of phenylpropanoids. Plant Physiol Biochem. 2008; 46(3): 356–370. 10.1016/j.plaphy.2007.12.009 18272377 PMC2860624

[24] Inouye S. Firefly luciferase: an adenylate-forming enzyme for multicatalytic functions. Cell Mol Life Sci. 2010; 67: 387–404. 10.1007/s00018-009-0170-8 19859663 PMC11115821

[25] Nakatsu T, Ichiyama S, Hiratake J, Saldanha A, Kobashi N, Sakata K, et al Structural basis for the spectral difference in luciferase bioluminescence. Nature. 2006; 440(7082): 372–376. 10.1038/nature04542 16541080

[26] Nakamura M, Maki S, Amano Y, Ohkita Y, Niwa K, Hirano T, et al Firefly luciferase exhibits bimodal action depending on the luciferin chirality. Biochem Biophys Res Commun. 2005; 331: 471–475. 10.1016/j.bbrc.2005.03.202 15850783

[27] Oba Y, Ojika M, Inouye S. Firefly luciferase is a bifunctional enzyme: ATP-dependent monooxygenase and a long chain fatty acyl-CoA synthetase. FEBS Lett. 2003; 540(1–3): 251–254. 12681517 10.1016/s0014-5793(03)00272-2

[28] Oba Y, Iida K, Inouye S. Functional conversion of fatty acyl-CoA synthetase to firefly luciferase by site-directed mutagenesis: a key substitution responsible for luminescence activity. FEBS Lett. 2009; 583(12): 2004–2008. 10.1016/j.febslet.2009.05.018 19450587

[29] Hastings JW. Biological diversity, chemical mechanisms, and the evolutionary origins of bioluminescent systems. J Mol Evol. 1983; 19(5): 309–321. 6358519 10.1007/BF02101634

[30] Widder EA. Bioluminescence in the ocean: origins of biological, chemical, and ecological diversity. Science. 2010; 328(5979): 704–708. 10.1126/science.1174269 20448176

[31] Drake EJ, Nicolai DA, Gulick AM. Structure of the EntB multidomain nonribosomal peptide synthetase and functional analysis of its interaction with the EntE adenylation domain. Chem Biol. 2006; 13(4): 409–419. 10.1016/j.chembiol.2006.02.005 16632253

[32] Dieckmann R, Neuhof T, Pavela-Vrancic M, von Döhren H. Dipeptide synthesis by an isolated adenylate-forming domain of non-ribosomal peptide synthetases (NRPS). FEBS Lett. 2001; 498(1): 42–45. 11389895 10.1016/s0014-5793(01)02471-1

[33] Drake EJ, Duckworth BP, Neres J, Aldrich CC, Gulick AM. Biochemical and structural characterization of bisubstrate inhibitors of BasE, the self-standing nonribosomal peptide synthetase adenylate-forming enzyme of acinetobactin synthesis. Biochemistry. 2010; 49(43): 9292–9305. 10.1021/bi101226n 20853905 PMC2964879

[34] Strieker M, Tanović A, Marahiel MA. Nonribosomal peptide synthetases: structures and dynamics. Curr Opin Struct Biol. 2010; 20(2): 234–240. 10.1016/j.sbi.2010.01.009 20153164

[35] González O, Ortíz-Castro R, Díaz-Pérez C, Díaz-Pérez AL, Magaña-Dueñas V, López-Bucio J, et al Non-ribosomal Peptide Synthases from *Pseudomonas aeruginosa* Play a Role in Cyclodipeptide Biosynthesis, Quorum-Sensing Regulation, and Root Development in a Plant Host. Microb Ecol. 2017; 73(3): 616–629. 10.1007/s00248-016-0896-4 27900439

[36] Arora P, Goyal A, Natarajan VT, Rajakumara E, Verma P, Gupta R, et al Mechanistic and functional insights into fatty acid activation in *Mycobacterium tuberculosis*. Nat Chem Biol. 2009; 5(3): 166–173. 10.1038/nchembio.143 19182784 PMC2644305

[37] Zhang Z, Zhou R, Sauder JM, Tonge PJ, Burley SK, Swaminathan S. Structural and functional studies of fatty acyl adenylate ligases from *E*. *coli* and *L*. *pneumophila*. J Mol Biol. 2011; 406(2): 313–324. 10.1016/j.jmb.2010.12.011 21185305 PMC3040979

[38] Freas N, Newton P, Perozich J. Analysis of Nucleotide Diphosphate Sugar Dehydrogenases reveals family and group-specific relationships. FEBS Open Bio. 2016; 6(1): 77–89. 10.1002/2211-5463.12022 27047744 PMC4794789

[39] Irvin J, Ropelewski A, Perozich J. *In silico* analysis of heme oxygenase structural homologues identifies group-specific conservations. FEBS Open Bio. 2017; 7: 1480–1498. 10.1002/2211-5463.12275 28979838 PMC5623701

[40] Altschul SF, Madden TL, Schäffer AA, Zhang J, Zhang Z, Miller W, et al Gapped BLAST and PSI-BLAST: a new generation of protein database search programs. Nucleic Acids Res. 1997; 25: 3389–3402. 9254694 10.1093/nar/25.17.3389 PMC146917

[41] Notredame C, Higgins H. T-coffee: A Novel Method for Fast and Accurate Multiple Sequence Alignment. J Mol Biol. 2000; 302: 205–217. 10.1006/jmbi.2000.4042 10964570

[42] Ilinkin I, Ye J, Janardan R. Multiple structure alignment and consensus identification for proteins. BMC Bioinformatics. 2010; 11: 71 10.1186/1471-2105-11-71 20122279 PMC2829528

[43] Prlić A, Bliven S, Rose PW, Bluhm WF, Bizon C, Godzik A, et al Pre-calculated protein structure alignments at the RCSB PDB website. Bioinformatics. 2010; 26: 2983–2985. 10.1093/bioinformatics/btq572 20937596 PMC3003546

[44] Ye Y, Godzik A. Flexible structure alignment by chaining aligned fragment pairs allowing twists. Bioinformatics. 2003; 19(suppl.2): ii246–ii255. 14534198 10.1093/bioinformatics/btg1086

[45] Nicholas KB, Nicholas HB, Deerfield DW. GeneDoc: Analysis and Visualization of Genetic Variation. EMB NEWS. 1997; 4: 14.

[46] Sayle A, Milner-White EJ. RasMol: Biomolecular graphics for all. Trends Biochem Sci. 1995; 20: 374–376. 7482707 10.1016/s0968-0004(00)89080-5

[47] Pettersen EF, Goddard TD, Huang CC, Couch GS, Greenblatt DM, Meng EC, et al UCSF Chimera–a visualization system for exploratory research and analysis. J Comput Chem.2004; 25(13):1605–1612. 10.1002/jcc.20084 15264254

[48] Chen VB, Arendall WB, Headd JJ, Keedy DA, Immormino RM, Kapral GJ, et al MolProbity: all-atom structure validation for macromolecular crystallography. Acta Crystallogr D Biol Crystallogr. 2010; 66(Pt 1): 12–21. 10.1107/S0907444909042073 20057044 PMC2803126

[49] Bailey TL, Elkan C. Fitting a mixture model by expectation maximization to discover motifs in biopolymers. ISMB. 1994; 2: 28–36. 7584402

[50] Bailey TL, Gribskov M. Combining evidence using p-values: application to sequence homology searches. Bioinformatics. 1998; 14: 48–54. 9520501 10.1093/bioinformatics/14.1.48

[51] Hempel J, Perozich J, Wymore T, Nicholas H. An algorithm for identification and ranking of family-specific residues, applied to the ALDH3 family. Chemico-Biological Interactions. 2003; 143–144: 23–28. 12604185 10.1016/s0009-2797(02)00165-5

[52] Lichtarge O, Bourne HR, Cohen FE. An evolutionary trace method defines binding surfaces common to protein families. J Mol Biol. 1996; 257(2): 342–358. 10.1006/jmbi.1996.0167 8609628

[53] Innis CA, Shi J, Blundell TL. Evolutionary trace analysis of TGF-beta and related growth factors: implications for site-directed mutagenesis. Protein Eng. 2000; 13(12): 839–847. 11239083 10.1093/protein/13.12.839

[54] Capra JA, Singh M. Predicting functionally important residues from sequence conservation. Bioinformatics 2007; 23(15): 1875–1882. 10.1093/bioinformatics/btm270 17519246

[55] Felsenstein J. PHYLIP manual, Version 3.3 Berkeley: University of California, University Herbarium 1990.

[56] Capella-Gutiérrez S, Silla-Martínez JM, Gabaldón T. TrimAl: a tool for automated alignment trimming in large-scale phylogenetic analyses. Bioinformatics. 2009; 25: 1972–1973. 10.1093/bioinformatics/btp348 19505945 PMC2712344

[57] Glaser F, Pupko T, Paz I, Bell RE, Bechor-Shental D, Martz E, et al ConSurf: identification of functional regions in proteins by surface-mapping of phylogenetic information. Bioinformatics. 2003; 19: 163–164. 12499312 10.1093/bioinformatics/19.1.163

[58] Laskowski RA, Swindells MB. LigPlot+: multiple ligand-protein interaction diagrams for drug discovery. J Chem Inf Model. 2011; 51: 2778–2786. 10.1021/ci200227u 21919503

[59] Gocht M, Marahiel MA. Analysis of core sequences in the D-Phe activating domain of the multifunctional peptide synthetase TycA by site-directed mutagenesis. J Bacteriol. 1994; 176: 2654–2662. 8169215 10.1128/jb.176.9.2654-2662.1994 PMC205405

[60] Black PN, Zhang Q, Weimar JD, DiRusso CC. Mutational analysis of a fatty acyl-coenzyme A synthetase signature motif identifies seven amino acid residues that modulate fatty acid substrate specificity. J Biol Chem. 1997; 272(8): 4896–4903. 9030548 10.1074/jbc.272.8.4896

[61] Khare G, Gupta V, Gupta RK, Gupta R, Bhat R, Tyagi AK. Dissecting the role of critical residues and substrate preference of a Fatty Acyl-CoA Synthetase (FadD13) of *Mycobacterium tuberculosis*. PLoS One. 2009;4(12): e8387 10.1371/journal.pone.0008387 20027301 PMC2793005

[62] Weimar JD, DiRusso CC, Delio R, Black PN. Functional role of fatty acyl-coenzyme A synthetase in the transmembrane movement and activation of exogenous long-chain fatty acids. Amino acid residues within the ATP/AMP signature motif of *Escherichia coli* FadD are required for enzyme activity and fatty acid transport. J Biol Chem. 2002; 277(33): 29369–29376. 10.1074/jbc.M107022200 12034706

[63] Koksharov MI, Ugarova NN. Strategy of mutual compensation of green and red mutants of firefly luciferase identifies a mutation of the highly conservative residue E457 with a strong red shift of bioluminescence. Photochem Photobiol Sci. 2013; 12(11): 2016–2027. 10.1039/c3pp50242b 24057044

[64] Modestova Y, Koksharov MI, Ugarova NN. Point mutations in firefly luciferase C-domain demonstrate its significance in green color of bioluminescence. Biochim Biophys Acta. 2014; 1844(9): 1463–1471. 10.1016/j.bbapap.2014.04.021 24802181

[65] Perozich J, Nicholas H, Wang BC, Lindahl R, Hempel J. Relationships within the aldehyde dehydrogenase extended family. Protein Sci. 1999; 8: 137–146. 10.1110/ps.8.1.137 10210192 PMC2144113

[66] Jörnvall H. Differences between alcohol dehydrogenases. Eur J Biochem. 1977; 72: 443–452. 320001 10.1111/j.1432-1033.1977.tb11268.x

[67] Persson B, Krook M, Jörnvall H. Characteristics of short-chain alcohol dehydrogenases and related enzymes. Eur J Biochem. 1991; 200: 537–543. 1889416 10.1111/j.1432-1033.1991.tb16215.x

[68] Perozich J, Hempel J, Morris SM. Roles of conserved residues in the arginase family. Biochim Biophys Acta. 1998; 1382(1): 23–37. 9507056 10.1016/s0167-4838(97)00131-3

[69] Nelson DL, Cox MM. Lehninger Principles of Biochemistry, 7^th^ edition New York: WH Freeman; 2017.

[70] Marahiel MA, Stachelhaus T, Mootz HD. Modular Peptide Synthetases Involved in Nonribosomal Peptide Synthesis. Chem Rev. 1997; 97(7): 2651–2674. 11851476 10.1021/cr960029e

[71] Youn B, Camacho R, Moinuddin SG, Lee C, Davin LB, Lewis NG, et al Crystal structures and catalytic mechanism of the Arabidopsis cinnamyl alcohol dehydrogenases AtCAD5 and AtCAD4. Org Biomol Chem. 2006; 4(9): 1687–1697. 10.1039/b601672c 16633561

[72] Ropelewski AJ, Nicholas HB, Gonzalez Mendez RR. MPI-PHYLIP: parallelizing computationally intensive phylogenetic analysis routines for the analysis of large protein families. PLoS One. 2010; 5: e13999 10.1371/journal.pone.0013999 21085574 PMC2981553

[73] Branchini BR, Southworth TL, Fontaine DM, Murtiashaw MH, McGurk A, Talukder MH, et al Cloning of the Orange Light-Producing Luciferase from *Photinus scintillans*-A New Proposal on how Bioluminescence Color is Determined. Photochem Photobiol. 2017; 93(2): 479–485. 10.1111/php.12671 27861940

[74] Adams ST Jr, Mofford DM, Reddy GS, Miller SC. Firefly Luciferase Mutants Allow Substrate-Selective Bioluminescence Imaging in the Mouse Brain. Angew Chem Int Ed Engl. 2016; 55(16): 4943–4946. 10.1002/anie.201511350 26991209 PMC4972182

[75] Nishiguchi T, Yamada T, Nasu Y, Ito M, Yoshimura H, Ozawa T. Development of red-shifted mutants derived from luciferase of Brazilian click beetle Pyrearinus termitilluminans. J Biomed Opt. 2015t;20(10): 101205 10.1117/1.JBO.20.10.101205 26313214

[76] Ford TJ, Way JC. Enhancement of *E*. *coli* acyl-CoA synthetase FadD activity on medium chain fatty acids. Peer J. 2015; 3: e1040 10.7717/peerj.1040 26157619 PMC4493641

[77] Stevens BW, Lilien RH, Georgiev I, Donald BR, Anderson AC. Redesigning the PheA domain of gramicidin synthetase leads to a new understanding of the enzyme's mechanism and selectivity. Biochemistry. 2006; 45(51): 15495–15504. 10.1021/bi061788m 17176071

[78] Han JW, Kim EY, Lee JM, Kim YS, Bang E, Kim BS. Site-directed modification of the adenylation domain of the fusaricidin nonribosomal peptide synthetase for enhanced production of fusaricidin analogs. Biotechnol Lett. 2012; 34(7): 1327–1334. 10.1007/s10529-012-0913-8 22450515

[79] Hughes AJ, Keatinge-Clay A. Enzymatic extender unit generation for in vitro polyketide synthase reactions: structural and functional showcasing of *Streptomyces coelicolor* MatB. Chem Biol. 2011; 18(2): 165–176. 10.1016/j.chembiol.2010.12.014 21338915

[80] Le NH, Molle V, Eynard N, Miras M, Stella A, Bardou F, et al Ser/Thr Phosphorylation Regulates the Fatty Acyl-AMP Ligase Activity of FadD32, an Essential Enzyme in Mycolic Acid Biosynthesis. J Biol Chem. 2016; 291(43): 22793–22805. 10.1074/jbc.M116.748053 27590338 PMC5077212

[81] Liu Z, Ioerger TR, Wang F, Sacchettini JC. Structures of *Mycobacterium tuberculosis* FadD10 protein reveal a new type of adenylate-forming enzyme. J Biol Chem. 2013; 288(25): 18473–18483. 10.1074/jbc.M113.466912 23625916 PMC3689989

[82] Harwood KR, Mofford DM, Reddy GR, Miller SC. Identification of mutant firefly luciferases that efficiently utilize aminoluciferins. Chem Biol. 2011; 18(12): 1649–57. 10.1016/j.chembiol.2011.09.019 22195567 PMC3273327

