# Supplementary figures and images for: In silico analysis of class I adenylate-forming enzymes reveals family and group-specific conservations
Source: PLoS One. 2018 Sep 4;13(9):e0203218. doi: 10.1371/journal.pone.0203218 (PMC6122825; doi:10.1371/journal.pone.0203218)

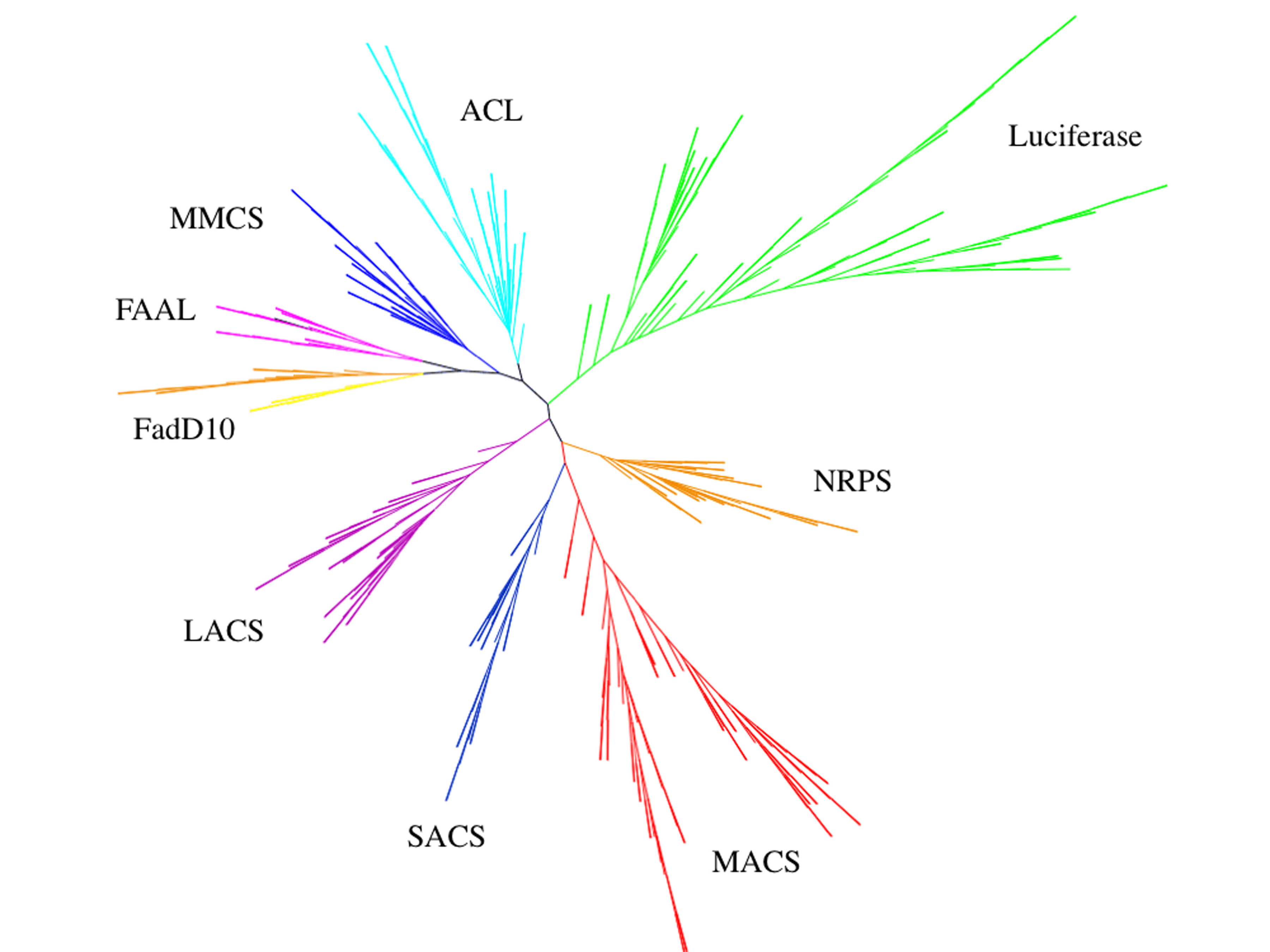

Supplement: S1 Fig — Branches are color-coded based on enzyme type: green = luciferases, purple = LACS, cyan = ACL, blue = MMCS, pink = FAAL, orange = NRPS, yellow = FadD10, navy = SACS and red = MACS. (TIF) [file pone.0203218.s002.tif]
